# Towards integrated photonic interposers for processing octave-spanning microresonator frequency combs

**DOI:** 10.1038/s41377-021-00549-y

**Published:** 2021-05-26

**Authors:** Ashutosh Rao, Gregory Moille, Xiyuan Lu, Daron A. Westly, Davide Sacchetto, Michael Geiselmann, Michael Zervas, Scott B. Papp, John Bowers, Kartik Srinivasan

**Affiliations:** 1 Physical Measurement Laboratory, Microsystems and Nanotechnology Division, National Institute of Standards and Technology, Gaithersburg, MD 20899 USA; 2 Maryland NanoCenter, University of Maryland, College Park, 20742 MD USA; 3 Joint Quantum Institute, NIST/University of Maryland, College Park, MD 20742 USA; 4 Ligentec, EPFL Innovation Park, Batiment C, Lausanne, Switzerland; 5 Physical Measurement Laboratory, Time and Frequency Division, National Institute of Standards and Technology, Boulder, CO 80305 USA; 6 Department of Physics, University of Colorado, Boulder, CO 80309 USA; 7 Department of Electrical and Computer Engineering, University of California, Santa Barbara, CA 93106 USA

**Keywords:** Frequency combs, Nonlinear optics, Integrated optics

## Abstract

Microcombs—optical frequency combs generated in microresonators—have advanced tremendously in the past decade, and are advantageous for applications in frequency metrology, navigation, spectroscopy, telecommunications, and microwave photonics. Crucially, microcombs promise fully integrated miniaturized optical systems with unprecedented reductions in cost, size, weight, and power. However, the use of bulk free-space and fiber-optic components to process microcombs has restricted form factors to the table-top. Taking microcomb-based optical frequency synthesis around 1550 nm as our target application, here, we address this challenge by proposing an integrated photonics interposer architecture to replace discrete components by collecting, routing, and interfacing octave-wide microcomb-based optical signals between photonic chiplets and heterogeneously integrated devices. Experimentally, we confirm the requisite performance of the individual passive elements of the proposed interposer—octave-wide dichroics, multimode interferometers, and tunable ring filters, and implement the octave-spanning spectral filtering of a microcomb, central to the interposer, using silicon nitride photonics. Moreover, we show that the thick silicon nitride needed for bright dissipative Kerr soliton generation can be integrated with the comparatively thin silicon nitride interposer layer through octave-bandwidth adiabatic evanescent coupling, indicating a path towards future system-level consolidation. Finally, we numerically confirm the feasibility of operating the proposed interposer synthesizer as a fully assembled system. Our interposer architecture addresses the immediate need for on-chip microcomb processing to successfully miniaturize microcomb systems and can be readily adapted to other metrology-grade applications based on optical atomic clocks and high-precision navigation and spectroscopy.

## Abstract (web summary)

Key steps towards photonic interposers for integrated optical synthesizers are shown: filters, mixers, linear processing of an octave-spanning microcomb, integration of microcomb and interposer layers, and a system-level feasibility analysis.

## Graphical abstract

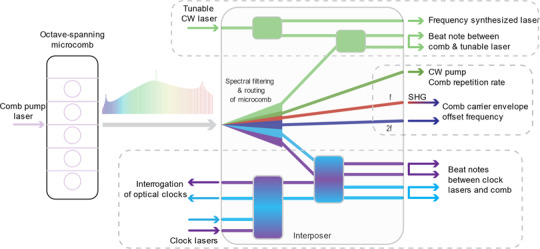

## Introduction

Optical microcombs, generated in micro and nanophotonic resonators, have substantially broadened the reach of applications of optical frequency combs^1^. Along with the promise of a dramatic transformation from traditional table-top and rack-mount form factors to chip-scale integrated systems, a variety of applications have been shown to benefit from the use of microcombs^2–4^. Furthermore, persistent innovation enabled by the precision nanofabrication of nanophotonic resonators continues to yield desirable and exotic optical microcombs^5–9^ for next-generation systems. The convergence of nanophotonic resonators with scalable integrated photonics inherently supports the promise of creating integrated microcomb-based systems, with immediate applications in optical frequency synthesis^10–12^, optical atomic clocks^13,14^, optical distance ranging^15–17^, optical spectroscopy^18–20^, microwave and radiofrequency photonics^21–23^, astronomy^24,25^, and telecommunications^26–28^.

However, to realize these integrated microcomb-based systems, integrated photonic interposers that connect and operate on optical signals that transit between the many constituent photonic components will be critical. In fact, the pursuit of such integrated systems has driven recent progress in active photonics, e.g., lasers^29–32^ and detectors^33^, nonlinear photonics in microresonators^5–9,34,35^ and waveguides^36–39^, and passive photonics and heterogeneous integration^40–42^, and has motivated milestones such as the generation of microcombs using chip-scale lasers^43–45^. Photonic interposers that collect, filter, route, and interface light between many such active and passive devices are essential to realize the improvements in cost, size, weight, and power, performance, and scalability, offered by microcombs and integrated photonics, and will promote further system-level innovation using frequency combs. Such interposers need to integrate multiple broadband high-performance photonic elements, manage octave-wide light, and maintain modal and polarization purity in a low-loss and high damage threshold photonics platform while pragmatically balancing heterogeneous integration and chip-to-chip coupling on a system-level architecture.

In this work, we consider integrated photonic interposers in the context of optical frequency synthesis. Optical frequency synthesis is one application in which the transition from lab-scale instrumentation to deployable technology hinges on the ability to combine microcomb technology with other integrated photonics. Optical frequency synthesizers generate stable, accurate, and precise optical frequencies from a standard microwave reference, have traditionally used mode-locked solid-state and fiber lasers to derive a fully stabilized self-referenced frequency comb^10,11^, and are indispensable in frequency metrology and timekeeping^13,46^, coherent light detection and ranging^15^, spectroscopy^18^, microwave synthesis^21^, and astronomy^24^. Yet, the cost and size of such table-top systems have limited their widespread application.

While substantial progress has been made recently towards optical frequency synthesis using integrated photonic devices^12,47–51^, these nascent efforts have required the use of free-space and fiber-optic components that hinders the overall goal of having standalone chip-size microcomb systems. These efforts have employed microcombs in on-chip silicon nitride (Si_3_N_4_) and silica microresonators^12,47^ and bulk crystalline resonators^48^, supercontinuum and second harmonic generation (SHG) in nonlinear silicon-on-insulator waveguides^51^, and phase locking in indium phosphide photonic integrated circuits^48,49^. Each of these photonic platforms offers different devices and functionality that are beneficial to building an integrated optical frequency synthesizer.

Here, we propose an integrated photonics interposer architecture for a microcomb-based optical frequency synthesizer that collects, routes, and interfaces broadband light from discrete chiplets and heterogeneously integrated photonic devices. We experimentally demonstrate the constituent passive elements of the proposed interposer, i.e., octave-wide dichroic couplers, resonant filters, and multimode interferometers (MMIs), and confirm that their performance agrees with our electromagnetic simulations via short-loop tests. The remaining heterogeneous integration-based components have been reported elsewhere previously^33,39,42^. We use the Si_3_N_4_ photonic platform, based on requirements of low absorption, high damage threshold, and broad optical transparency. We directly verify the suitability of the dichroics to process octave-wide light by using an octave-spanning microcomb generated in a thick Si_3_N_4_ chip as the input. Subsequently, we demonstrate the octave-wide spectral processing of an octave-spanning microcomb, key to the interposer, via an integrated sequence of the dichroic couplers and a tunable ring filter, measuring spectral contrast between the optical bands of interest that is appropriate for our intended application and congruent with our short-loop characterization of the individual components. Further, we report the single-chip integration of a broadband Si_3_N_4_ microcomb generated in a thick Si_3_N_4_ layer with the thinner Si_3_N_4_ photonic layer used for the interposer components, demonstrating a route towards additional system-level consolidation. Finally, we numerically confirm the feasibility of our proposed scheme for an integrated photonics interposer for frequency synthesis through a detailed system-level analysis, calculating the signal-to-noise ratios (SNRs) for the expected constituent beat notes based on the experimentally demonstrated performance of the different components.

**Fig. 1:**
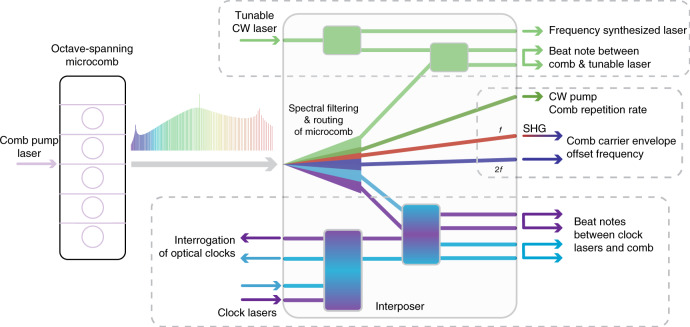
Concept of photonic interposers for integrated processing of microcombs. Photonic interposers for fully integrated microcomb-based systems will need to interface multiple photonic devices, such as microcombs and other nonlinear elements, and lasers and photodetectors. The functions of such interposers can be broadly classified into two parts, first, the broadband spectral routing of microcombs, and second, the coherent mixing of specific filtered bands and teeth of the microcombs with additional external signals. The broadband spectral routing of microcombs includes separation of *f* and 2*f* components for self-referencing via second harmonic generation (SHG) and additional filtering for repetition rate detection, which together enable microcomb stabilization for metrological-grade applications. Depending on the application, further microcomb processing may be required, such as extraction of the pertinent comb reference band for optical frequency synthesis or of comb teeth matched to specific atomic transitions for optical clocks. These bands and teeth are subsequently mixed with tunable lasers and clock lasers for beat note detection for synthesis and timekeeping. In this work, we demonstrate individual passive components suitable for such an interposer for a dual-microcomb-based optical frequency synthesizer and implement the requisite spectral filtering of an octave-spanning microcomb

Figure 1 schematically depicts microcombs and other integrated photonic devices in the context of systems such as optical frequency synthesizers and optical atomic clocks. To transition to an integrated system from the table-top, numerous optical functions are required with the simultaneous operation of multiple photonic devices in lockstep. These functions nominally translate to different material requirements—optical gain is required for lasers, *χ*^(3)^ nonlinearity for microcombs, *χ*^(2)^ nonlinearity for SHG, low linear loss for passives, and a high responsivity, low dark current material for photodetectors. To address this challenge of combining multiple material responses and platforms, one approach is to interface several chiplets of different photonic materials on a common carrier via chip-to-chip facet coupling, benefiting from the use of reliable well-established photonics, and the ability to prequalify each photonic element prior to system assembly. Another approach is to integrate all functions and materials together on one main photonic chip, akin to heterogeneous integration, where the benefits inherent to having a system on a chip will come at the cost of the requisite research and development. Crucially, a judicious combination of chip-to-chip facet coupling and heterogeneous integration can balance the pragmatism of using discrete chiplets of well-established photonic elements with the benefits and cost of heterogeneously integrating multiple material systems together, using a photonic interposer to bind the system together.

Figure 1 also indicates the nature of microcomb processing required of such photonic interposers. Spectral bands of combs generated in nonlinear resonators pumped by chip-scale lasers need to be adequately filtered across an octave bandwidth to facilitate stabilization via *f*–2*f* self-referencing, where additional nonlinear devices are required for the frequency doubling. In addition, narrow spectral filtering of the strong pumps that drive the microcombs is required to prevent damage to and maintain the performance of both slow and fast photodetectors that monitor optical power and facilitate phase locking via optical interference in on-chip coherent mixers. The approach upon which our interposer design is based uses two phase-stable interlocking Kerr combs to form the optical reference for synthesis^12^, each pumped near 1550 nm, and generated in separate Si_3_N_4_ and Si_3_N_4_ or silicon dioxide (SiO_2_) microresonators with repetition rates of ≈1 THz and ≈20 GHz, respectively. The dual-microcomb system assists in reducing power consumption compared to a single octave-spanning microcomb of a directly detectable repetition rate, where the octave-spanning Si_3_N_4_ comb is used for self-referencing and a narrower 20 GHz comb is used for repetition rate and synthesis frequency detection.

## Results

### Interposer architecture

**Fig. 2:**
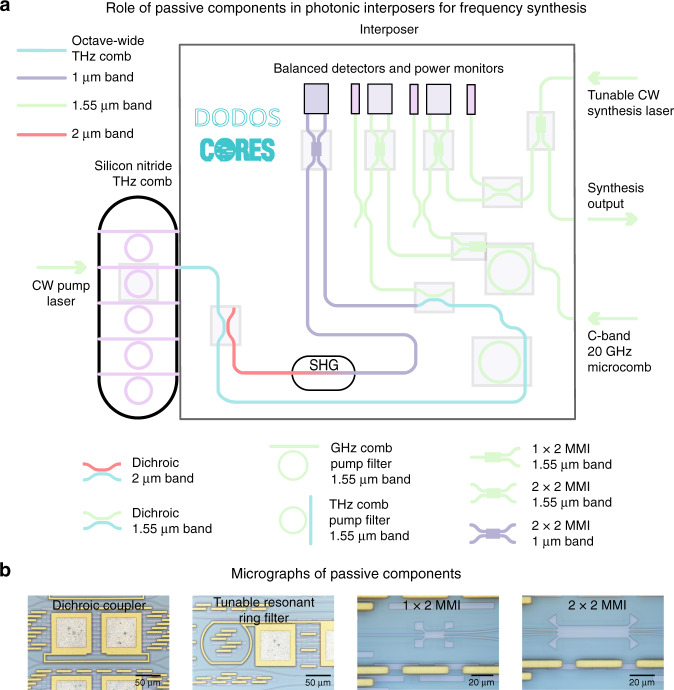
Envisioned role of passive components within a photonic interposer for a dual-microcomb-based optical frequency synthesizer. **a** Conceptual schematic showing how the passive components demonstrated in this work (highlighted in light gray), octave-wide dichroics, tunable ring filters for microcomb pump extinction, and multimode interferometers, could fit into the proposed interposer and system architecture to form an integrated dual-microcomb-based frequency synthesizer. The interposer is interfaced with THz repetition rate silicon nitride (also highlighted in light gray) and 20 GHz repetition rate silicon nitride or silica microcombs and a tunable laser via facet coupling, and with photodetectors and a second harmonic frequency doubler via heterogeneous integration. Dichroic directional couplers spectrally filter the silicon nitride microcomb in preparation for self-referencing and interference with the 20 GHz microcomb for repetition rate stabilization. In turn, the output tunable frequency synthesis laser is referenced to the 20 GHz microcomb. Multimode interferometers are utilized to generate these stabilization beat notes via balanced detection, and power monitors are used for additional system-level monitoring. Metal traces are not shown in this schematic. A detailed discussion showing the feasibility of such a system using only integrated components is included in the Supplementary information (Notes 7 and 8). **b** Micrographs of the individual interposer components shown in this work, dichroic directional couplers, ring resonator tunable filters, and multimode interferometers

Figure 2a shows a schematic of the full photonic interposer design, which is based on transverse-electric polarized guided light in a 400-nm-thick stoichiometric Si_3_N_4_ photonic platform with upper and lower SiO_2_ cladding. The Si_3_N_4_ platform is well established for numerous applications, and its low optical loss and high optical damage threshold, coupled with its broad optical transparency, assist in processing both low- and high-power optical signals across the octave bandwidth. The nitride film thickness and waveguide widths are chosen to balance optical confinement, proximity to the optical single-mode condition, and coupling to both heterogeneously integrated and facet-coupled elements, in contrast to microcombs where the anomalous dispersion required for octave-spanning bright Kerr solitons necessitates films that are nearly a factor of two thicker. Further details regarding optical confinement and the number of modes can be found in the Supplementary information (Note 1).

### Interposer components

The passive components of the interposer are dichroic directional couplers (hereafter referred to as dichroics), resonant filters, 50:50 MMIs, and power splitters and taps that operate on the two microcombs and the tunable synthesis laser (Fig. 2b). These elements interface with a frequency doubler (SHG) including a polarization rotator and a photodetector array that are heterogeneously integrated^33,39,42,52^. The output of the octave-spanning Si_3_N_4_ comb chip is directed to two cascaded dichroics that spectrally filter the microcomb into three key spectral bands, a long and a short wavelength band near 2 and 1 μm, respectively, separated by an octave, and the center band near 1.55 μm. The first dichroic separates out light in the 2 μm band from shorter wavelengths, and the second dichroic separates 1.55 μm light from shorter wavelengths (in particular, the 1 μm light). The 2 μm light is led to the frequency doubler, after which the upconverted output in the 1 μm band is coherently mixed with the 1 μm microcomb light in a 2 × 2 50:50 MMI and detected to extract the carrier-envelope offset frequency of the THz comb. Two 1 × 2 50:50 MMIs split the 20 GHz comb and the tunable synthesis laser (which reside on separate chips that are butt coupled to the interposer). An additional 2 × 2 50:50 MMI is used to coherently mix the 20 GHz comb with the 1.55 μm band of the Si_3_N_4_ comb light, while a second 2 × 2 50:50 MMI mixes the 20 GHz comb with the tunable laser. The MMI outputs are used to phase lock the two microcombs and detect the precise optical frequency of the tunable laser. In addition, two thermally tunable microring resonators filter out the microcomb pumps in the 1.55 μm band, and power taps and detectors are used to monitor the optical power of the microcombs and tunable laser. In the following three subsections, we first demonstrate the individual passive components of the interposer, i.e., MMIs, ring filters, and octave-wide dichroics. Our choice of the specific passive devices here is motivated by their specific application. In particular, we use ring filters to filter microcomb pumps because of the inherent vernier effect with the remainder of the microcomb that minimizes any undesired filtering of other microcomb tones, the ability to engineer microring-waveguide coupling across the wide spectral bands used here, and the capability to thermally tune the ring filters to precisely overlap the pump frequencies. Similarly, our choice of directional couplers for the dichroics is motivated by their inherent low-loss and transmissive operation, along with the ability to design large bandwidths with high extinction ratios. We design these passive components employing a combination of waveguide eigenmode and 3D finite-difference time-domain (FDTD) simulations, and fabricate them on 100 mm wafers using process sequences based on both deep-ultraviolet lithography (Ligentec) and electron-beam lithography (NIST). We validate our designs and fabrication by experimentally confirming the predicted component performance using both continuous-wave (CW) light and octave-spanning microcomb light. Progress in the heterogeneously integrated interposer components, i.e., the frequency doubler and the photodetectors, has already been reported elsewhere^33,39,42,52^, and we do not develop them further here. These components are discussed in depth in the context of a system-level analysis later in the Supplementary information (Notes 7 and 8).

### Multimode interferometers

**Fig. 3:**
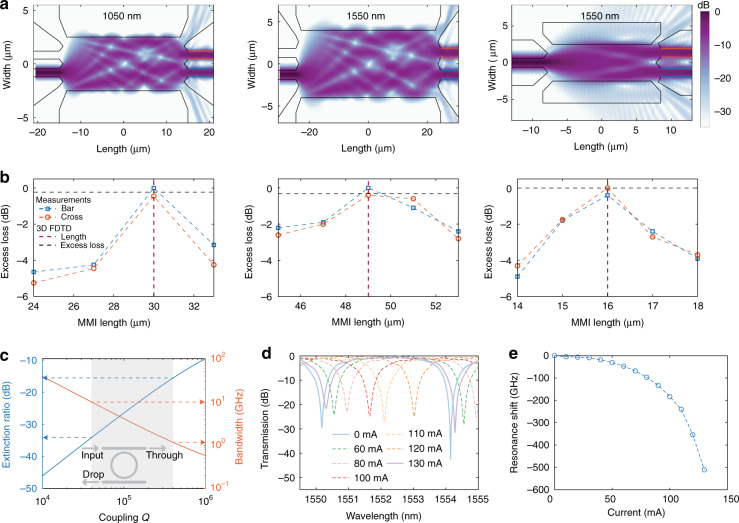
Multimode interferometers and microring filters. **a** Simulations showing the propagation of light from left to right in the three multimode interferometers at 1050 and 1550 nm. The corners of the butterfly geometry guide out light at the ≈−25 dB level, suppressing potential reflections. The bar and cross output ports are highlighted in orange and blue outlines, respectively. Cross-sections of |**E**(*x*,*y*,*z*)|2 are plotted with *z* set to half the height of the MMIs. **b** Corresponding continuous-wave measurements of the bar and cross ports of the MMIs for a range of MMI lengths. In each case, the optimal MMI length matches the predicted length from the simulations in (**a**). The associated measurement uncertainty is <0.2 dB based on one standard deviation in the transmission of five identical cascaded multimode interferometers. **c** Simulated dependence of microring filter characteristics, extinction ratio and bandwidth, on coupling *Q* for an intrinsic *Q* of 10^6^. Coupling *Q*s between 4 × 10^4^ and 4 × 10^5^ yield extinction ratios between 15 and 35 dB and corresponding filter bandwidths of 1–10 GHz, a range of filter characteristics suitable for our intended application of suppressing the pump of microcombs. **d** Measured transmission spectra for a thermally tuned microring filter using an integrated heater. **e** Variation of resonance frequency shift with heater current corresponding to (**d**), showing over one free spectral range of tuning

Figure 3a shows 3D FDTD simulations of the 1 × 2 and 2 × 2 50:50 MMIs that function as power splitters and coherent mixers, respectively (see “Methods” and Supplementary information (Note 2) for details). The transmission ratio of the optical powers at the output ports of the 2 × 2 MMIs impact the balanced detection of the beat notes for phase locking, motivating our choice of a butterfly MMI over a directional coupler. The corners of the butterfly geometry funnel out potential reflections that are deleterious to both the unity transmission ratio and the operation of an integrated circuit^53^. The corresponding CW transmission measurements of the bar and cross ports are shown in Fig. 3b for a range of MMI lengths, and the optimum MMI length agrees with our simulations. The excess loss, defined as transmission loss relative to the maximum transmission (nominally −3 dB), for all three optimal MMI lengths is <0.5 dB, and includes variations from coupling on and off the chip.

### Microring filters

The filter bandwidth and extinction ratio of the thermally tunable symmetric add-drop microring filters that filter CW pump light are determined by the intrinsic and coupling quality factors (*Q*), which depend on absorption and scattering, and on the magnitude of coupling between the bus waveguide and the microring^54,55^, respectively (Fig. 3c). Measurements (Fig. 3d, e) show that a ring filter with 50 μm radius (474.8 GHz free spectral range (FSR)) suitable for the Si_3_N_4_ microcomb (coupling *Q* ≈ 2 × 10^4^, intrinsic *Q* ≈ 10^6^) can be thermally tuned over 500 GHz, i.e., over an entire FSR, while maintaining adequate extinction, a requirement for matching the resonance of the filter with the pump of the Si_3_N_4_ microcomb. The maximum extinction measured, and variations therein, are limited by thermally induced perturbations to the coupling, and the polarization extinction ratio of the input light. For typical THz repetition rate microcombs, the pump power is 15–20 dB higher than the neighboring comb teeth. Therefore, to flatten the pump comb tooth to match the surrounding teeth, a coupling *Q* as high as ≈10^5^ can be adequate. A similar microring with coupling *Q* ≈ 10^5^ will be suitable for filtering the 20 GHz microcomb. Additional details regarding design and fabrication can be found in the Supplementary information (Note 3) and the “Methods.” While our intended application requires moderate filtering and can take advantage of an inherent vernier effect between the filter and microcomb resonators, more demanding applications can use cascaded ring filters to synthesize more complex filter responses^56,57^. The 474.8 GHz ring filter FSR is sufficiently close to half of the microcomb’s THz FSR for the vernier effect to ensure there is no spurious filtering of the THz microcomb in the C-band. Similarly, the 474.8 GHz FSR also provides a spurious-filtering free bandwidth of ~3.8 THz in the C-band for the 20 GHz microcomb.

### Dichroic couplers

**Fig. 4:**
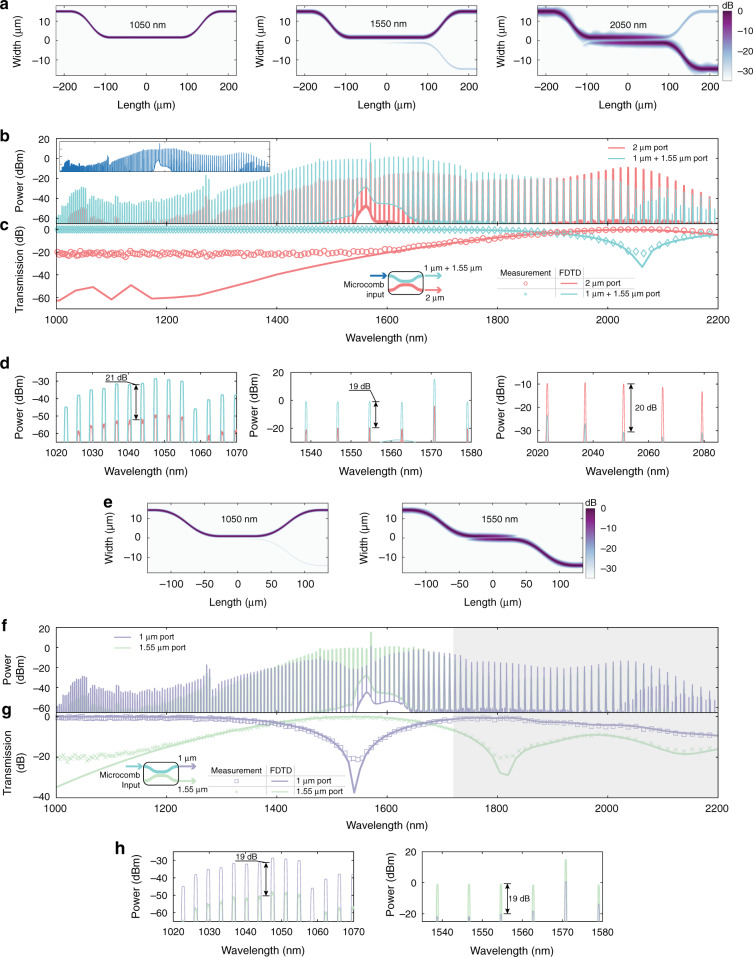
Octave-wide operation of dichroics. **a**–**d** First dichroic, whose purpose is to separate 2 μm light from shorter wavelengths. **a** Simulations at 1050, 1550, and 2050 nm showing extraction of the 2 μm band into the cross port. **b** Measured broadband experimental spectra at the bar and cross ports. The input is the microcomb shown in the inset. **c** Measured (symbols) and simulated (solid lines) octave-wide transfer function. At the cross or 2 μm port, extinction ratios of (21.4 ± 1.1) and (19.9 ± 0.8) dB are measured in the 1 and 1.55 μm bands, respectively. At the bar port, an extinction ratio of (18.1 ± 2.9) dB is measured in the 2 μm band. **d** Magnified individual spectral bands. **e**–**h** Second dichroic, whose purpose is to separate 1.55 μm light from shorter wavelengths. **e** Simulations at 1050 and 1550 nm, showing extraction of the 1.55 μm band into the cross port. **f** Measured broadband experimental spectra at the bar and cross ports. The input is the microcomb shown in the inset of (**b**)**. g** Measured (symbols) and simulated (solid lines) octave-wide transfer function. At the cross or 1.55 μm port, an extinction ratio of (20.1 ± 1.0) dB is measured in the 1 μm band, and at the bar port, an extinction ratio of (18.6 ± 3.3) dB is measured in the 1.55 μm band. **h** Magnified individual spectral bands. The performance of the dichroic in the spectral region shaded in (**f**, **g**) is relatively unimportant, as this region is filtered out by the first dichroic in the full interposer chip. In (**a**, **e**), cross-sections of |**E**(*x*,*y*,*z*)|2 are plotted with *z* set to half the height of the dichroics. The measured transfer functions shown in (**c**, **g**) are extracted from the corresponding transmission of the comb teeth in (**b**, **f**). The corresponding uncertainties reported in (**c**, **g**) correspond to line-to-line fluctuations in the measured comb spectra and include variations in coupling and are one standard deviation values

Figure 4a shows simulations for the dichroic that extracts the 2 μm microcomb band into the cross port. We measured the cross and bar port transmission for a range of directional coupler lengths using CW light at the three bands, and observed agreement with the expected optimized coupler length, with 15 dB of contrast at 2 and 1.55 μm, and over 30 dB at 1 μm, see the Supplementary information (Note 4) for details. Figure 4b shows the measured individual bar and cross port spectra of the optimized dichroic across the nominal octave bandwidth centered around the telecom C-band. The measurement uses an octave-spanning Si_3_N_4_ microcomb (Fig. 4b, inset), generated in a 770-nm-thick microring with low and broadband anomalous dispersion, as the input. Figure 4c compares the measured transmission with the simulated transmission, and magnified views of measurements in the 2, 1.55, and 1 μm bands are shown in Fig. 4d. Similarly, the second dichroic couples out the 1.55 μm microcomb light into the cross port, leaving the 1 μm band in the bar port, as seen in simulations at these wavelengths in Fig. 4e. Corresponding CW measurements indicated over 20 dB of contrast between the two ports, see the Supplementary information (Note 4) for details. The behavior of this dichroic in the 2 μm band is inconsequential because it is intended to process the Si_3_N_4_ microcomb after the 2 μm band is filtered out in the first dichroic (Fig. 2). Figure 4f shows the measured individual bar and cross port spectra of the optimized dichroic, using the same microcomb input employed to evaluate the first dichroic (Fig. 4b, inset). Figure 4g compares the simulated and measured transmission of the dichroic across the octave, and magnified views of the spectral bands are shown in Fig. 4h. Overall, the performance of the two dichroics is appropriate for our intended application and largely follows the simulated behavior, with deviations observed only below the ≈−20 dB level, likely originating from limitations of the measurement setup. Further details regarding design optimization and the experimental setup can be found in the “Methods” and Supplementary information (Notes 4 and 6).

### Integrated processing of an octave-spanning microcomb

**Fig. 5:**
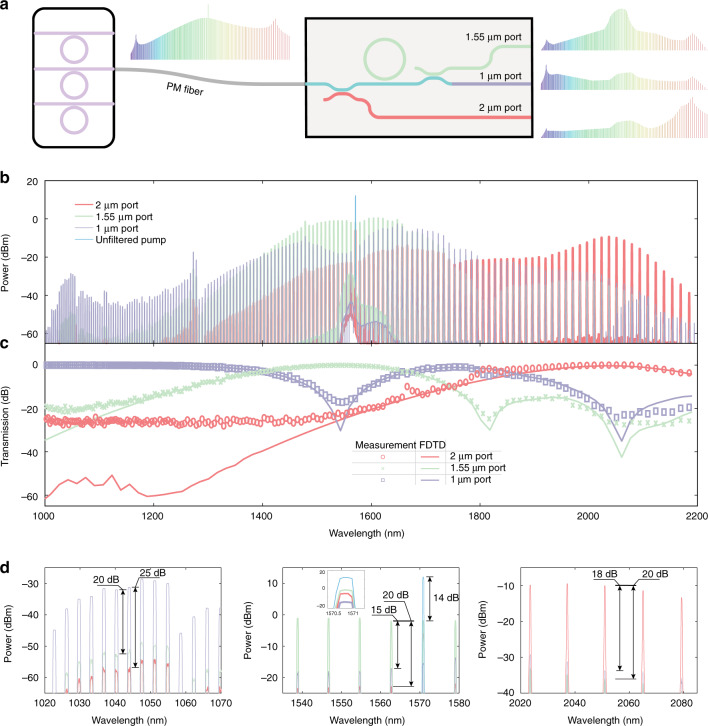
Integrated spectral processing of a microcomb. **a** Schematic for on-chip processing of a silicon nitride-based octave-spanning microcomb. PM: polarization maintaining. Here a PM fiber is used to link the two chips for convenience in testing, but finite element simulations suggest that direct facet-to-facet coupling with ~1 dB loss should be possible. **b** Experimental spectra measured at the three output ports. The microcomb shown in the inset of Fig. 4b is used as the input. **c** Measured (symbols) and simulated (solid lines) octave-wide transfer functions. The measured transfer function is extracted from the transmission of the comb teeth in (**b**). At the 1 μm port, extinction ratios of (16.2 ± 0.8) and (20.9 ± 2.2) dB are measured in the 1.55 and 2 μm bands, respectively. Similarly, at the 1.55 μm port, extinction ratios of (20.2 ± 0.7) and (25.6 ± 2.1) dB are measured in the 1 and 2 μm bands, and at the 2 μm port, extinction ratios of (26.1 ± 0.8) and (22.7 ± 0.9) dB are measured in the 1 and 1.55 μm bands. **d** Magnified comparison of the outputs at the three ports in the individual spectral bands. Separation of the three spectral bands into the three ports with 15–25 dB of contrast is observable, along with 14 dB of pump suppression after comb generation from the ring filter (light blue comb tooth). The uncertainties reported in (**c**) correspond to line-to-line fluctuations in the comb spectra and include variations in coupling, and are one standard deviation values

So far, we have presented the design and experimental characterization of individual interposer elements. As a first demonstration of processing an octave-spanning microcomb using a more integrated photonic chip that contains all of the aforementioned filtering capability, we measured the transmission through a chip comprised of a sequence of the two dichroics with a microring filter at the 1.55 μm band port (Fig. 5a), using an octave-spanning microcomb (Fig. 4b, inset) as the input. Measurements shown in Fig. 5b show that the three spectral bands of interest are routed into the three physical ports. The ring filter reduces the pump amplitude to that of the neighboring comb tones. Figure 5c compares the transfer function extracted from Fig. 5b to simulations based on 3D FDTD, excluding the effect of the ring filter that has no effect on the transmission envelope, showing good agreement between the two. Magnified views of the three spectral bands are shown in Fig. 5d. We observe 15–25 dB of extinction across the spectral bands at the outputs, along with 14 dB of pump suppression from the ring filter. Similar to the characterization of the individual dichroics, deviations occurring below the ≈−20 dB level result from limitations of the measurement; see the “Methods” and Supplementary information (Note 6) for more details regarding the fabrication and experimental setup.

### Towards microcomb-interposer integration

**Fig. 6:**
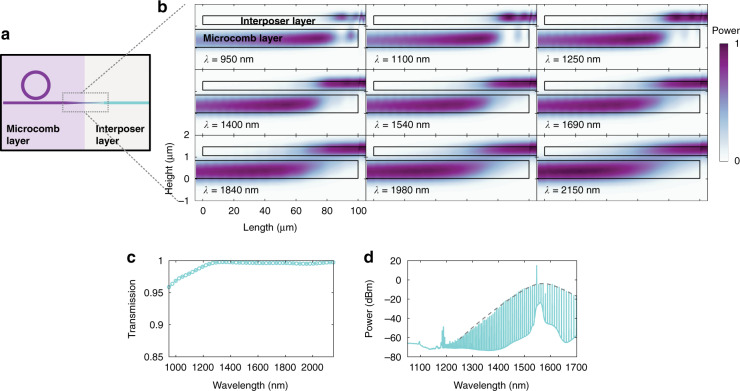
Towards microcomb-interposer integration. **a** Schematic showing integration of the microcomb and interposer photonics layers that are interfaced by a bilayer taper that can transfer an octave of comb bandwidth with negligible loss. **b**, **c** Simulations of a 100-μm-long bilayer taper showing low-loss broadband transfer of light. **d** Broadband microcomb, along with the corresponding sech^2^ fit, measured from a fabricated bilayer chip where the microcomb output is extracted through the bilayer taper into the interposer layer

Looking forward, we show that our microcomb sources can be integrated with our photonic interposer layer, as envisioned in Fig. 6a. Bright Kerr soliton generation directly within the 400-nm-thick Si_3_N_4_ interposer layer is not possible in conventional ring geometries due to the normal dispersion associated with all waveguide widths at that thickness. One could instead consider making the interposer out of a thicker Si_3_N_4_ layer (i.e., suitable for broadband anomalous dispersion), but the design of passive elements may be complicated by the increased confinement and larger numbers of modes supported by the thicker film. We instead adopt a dual-layer approach, shown in Fig. 6. Here, fabrication of a thick Si_3_N_4_ layer (the microcomb layer) is followed by a vertically coupled thin Si_3_N_4_ layer (the interposer layer), with chemical–mechanical polishing enabling control of the SiO_2_ film thickness separating the layers. A key challenge for this approach is the transfer of the microcomb to the interposer layer across a full octave of bandwidth. We address this challenge by using a 100 μm bilayer taper (schematic top view shown in Fig. 6a) that ensures adiabatic transfer of light with <1 dB of loss across an octave, simulated using 3D FDTD (Fig. 6b, c). The Si_3_N_4_ film thicknesses of the microcomb and interposer layers are 790 nm (a common thickness for broadband combs^17,26^) and 400 nm, respectively, with an interlayer SiO_2_ thickness of 200 nm (see Supplementary information (Note 5) for details). Both layers are tapered in width from 1 to 0.2 μm over a 100 μm length. Importantly, the adiabatic nature of the taper is such that it is relatively insensitive to precise interlayer SiO_2_ thickness (at the 50 nm level), as well as lateral offsets between the waveguide layers (at the 100 nm level). Figure 6d shows a Kerr soliton microcomb generated in a ring of 23 μm radius, measured after transfer through the bilayer taper. No spectral degradation was observed in comparison to a microcomb pumped in the opposite direction, where the microcomb does not pass through the bilayer taper. The reduced bandwidth of the microcomb compared to that used previously in this work precludes its use in the demonstration shown in Fig. 5, and stems from differences in dispersion that primarily arise from the different Si_3_N_4_ thickness used (790 nm targeted here vs. 770 nm previously). Nevertheless, this serves as a conclusive demonstration that the thick Si_3_N_4_ layer associated with microcomb generation can be integrated on the same chip with thinner Si_3_N_4_ that is preferable for linear functionality.

## Discussion

Different approaches have been established in the literature for integrated dichroic filtering. These include the use of symmetric and asymmetric directional couplers^58–60^, asymmetric Y-junctions^61–63^, sub-wavelength gratings^64,65^ and photonic crystals^66^, MMIs^67,68^, Mach–Zehnder interferometers^69^ and optical lattice filters^70^, and inverse designed structures^71^ on popular photonics platforms. Of these, the directional coupler-based approach is well suited for broadband applications such as ours here, having shown a combination of good extinction ratios, high bandwidths, low loss, and transmissive operation. Most pertinent to our work, bandwidths of over two-thirds of an octave^58^ and over an octave^59^, both centered around 1.55 µm, have been demonstrated, accompanied by losses varying between 0.5 and 3 dB and extinction ratios between 11 and 30 dB across the different bands of operation. Our dichroics, also based on directional couplers, are demonstrated over an octave of bandwidth, with losses <0.25 dB (measurements limited by variations in fiber coupling) and extinction ratios of 16–26 dB in the three pertinent bands (1, 1.55, and 2 µm). The performance offered by our other interposer components, MMIs and ring filters, is commensurate with the current state of the art in Si_3_N_4_ photonics^72–77^, where 0.5 dB of excess MMI loss, similar to our MMIs, and microring filters with intrinsic *Q*s ~ 10^6^ and extinction ratios in excess of 20 and 80 dB for first- and third-order filters have been reported. For the case of the ring filters, the utility of intrinsic *Q* and maximum extinction ratio is strongly application-dependent—for our application, we engineer the coupling *Q* to ensure strong undercoupling and overcoupling only up to a desired extent in the 1 and 1.55 µm bands, respectively. In the context of our bilayer taper microcomb source, much progress has been realized in multiplanar photonics using combinations of different photonic materials^29–33,39–42^, particularly in nonlinear photonics. Notably, linear high *Q* Si_3_N_4_ resonators have been previously integrated with silicon bus waveguides^78^. In relation to our proposed scheme here (Fig. 2), III–V-based SHG and photodetectors have been shown on insulators and 400-nm-thick Si_3_N_4_^33,39,42^.

We perform a numerical analysis to confirm the feasibility of the synthesizer proposed in Fig. 2. The SNRs of the three beat notes measured between the 2*f* and frequency-doubled *f* tones of the THz microcomb for the carrier-envelope offset frequency (*f*_CEO_) and self-referencing, between the dual microcombs for interlocking, and between the synthesis laser and the 20 GHz microcomb are key to the performance of such a system. The two beat notes of the dual microcombs and the synthesis laser-20 GHz microcomb lie within the nominal bandwidth for heterogeneously integrated photodetectors on Si_3_N_4_^33^. However, the carrier-envelope offset frequency (*f*_CEO_) beat note for self-referencing of the THz microcomb can in principle vary between −500 and +500 GHz (the repetition rate). By judicious tuning of the microring geometry, one can simultaneously achieve dual-dispersive waves at *f* and 2*f* frequencies along with the pinning of *f*_CEO_ to within the photodetector bandwidth. In particular, the microcomb dispersion is largely dominated by the microring cross-section (ring width and height), while the ring radius has comparatively minimal impact on the dispersion, and, therefore, appropriate choice of ring radius keeps *f*_CEO_ in a detectable range. Further details, including strategies for managing the *f*_CEO_ range for the bilayer integration approach indicated in Fig. 6, can be found in the Supplementary information (Note 8.1). In addition to interposer component performance, the beat note SNRs are determined by a combination of other photonics and electronics-related factors, such as chip laser power, microcomb performance, SHG efficiency, photodetector responsivity and bandwidth, transimpedance amplifier performance, coupling efficiencies, and transmission loss throughout the system, and locking electronics. The Supplementary information (Notes 7 and 8) offers a detailed discussion of the proposed system (using a silica microcomb for the 20 GHz comb), including the distribution of power throughout it, the impact of the aforementioned factors including interposer component performance, and the final SNRs of the three beat notes. Using a conservative analysis based on device performances corresponding to contemporary demonstrations and realistic system operation, we estimate the beat note SNRs as 16.9–25.5, 25, and 31.1 dB for *f*_CEO_, the dual-microcomb lock, and the synthesis laser-silica microcomb lock, adequate for system operation. Furthermore, improvements over the current performance of the interposer components shown here are seen to offer minimal improvement in the beat note SNRs; an analysis of the impact of dichroic extinction ratios and MMI excess losses is included in the Supplementary information (Note 8.9).

In summary, we have demonstrated octave-wide dichroic filters, MMIs, and tunable ring filters in the Si_3_N_4_ photonic platform. These passive elements are envisioned to be the core ingredients in a future integrated photonics interposer architecture for a microcomb-based optical frequency synthesizer that uses a variety of photonic devices to collect, route, and interface broadband light from discrete chiplets and heterogeneously integrated photonic devices. Such an architecture is important for addressing a key impediment in the full chip-scale integration of multiple material systems and functional responses for microcomb-based systems. We use the well-known Si_3_N_4_ photonic platform because of its low absorption, high damage threshold, and broad optical transparency, and validate our approach with a combination of electromagnetic calculations and measurements on fabricated devices integral to the interposer. We perform a series of short-loop tests where our designs for dichroic couplers, resonant filters, and MMIs show experimental performance well suited for processing microcombs, in congruence with our simulations. In addition to measurements using CW inputs, we use an octave-spanning microcomb generated in a thick Si_3_N_4_ chip as the input to directly confirm the ability of the dichroic couplers to process octave-wide light. Following the success of the individual interposer elements, we demonstrate octave-wide spectral processing of an octave-spanning microcomb through an integrated chain of two dichroic couplers and a ring filter, which constitute the key broadband comb processing sequence of the interposer, and measure the expected spectral contrast in the wavelength bands of interest, along with the flattening of the pump tone to match the remainder of the microcomb. Further, we report the single-chip integration of a broadband Si_3_N_4_ microcomb with the Si_3_N_4_ photonic layer used for the interposer components by using a broadband adiabatic taper to transfer the microcomb output between the thick microcomb and thinner interposer layers, indicating a path towards integrating microcombs with additional customizable photonic processing. Finally, we numerically analyze the potential performance of the proposed integrated photonics synthesizer architecture in light of the demonstrated component-level performance. The interposer components we have developed can be adapted to develop interposer architectures for other microcomb-based integrated systems for optical atomic clocks, high-precision spectroscopy, and precise navigation, among others, based on similar requirements for microcomb processing and system integration.

## Materials and methods

### Device designs

The devices are designed using a combination of eigenmode simulations, coupled-mode theory, and 3D FDTD simulations. Waveguide modes, microring modes, and effective indices are simulated across an octave bandwidth using COMSOL. Coupling coefficients between identical straight waveguides are determined through supermode simulations and the coupling between microrings and straight bus waveguides is calculated using coupled-mode theory. The propagation of light and related transmission transfer functions shown in Figs. 4–6 are extracted from octave-wide 3D FDTD simulations.

### Device fabrication

All devices used here are fabricated on SiO_2_-clad Si_3_N_4_ photonic platforms. Low-pressure chemical vapor deposition is used to deposit these Si_3_N_4_ layers. The Nanolithography Toolbox, a free package developed by the NIST Center for Nanoscale Science and Technology, was used for all device layouts. Broadband ellipsometry was used along with an extended Sellmeier model to evaluate the refractive index across the wavelength range of interest. All devices are fabricated on 100 mm silicon wafers. The octave-spanning microcomb, the interposer elements (MMIs, ring filters, and dichroics), and the bilayer microcomb are fabricated at Ligentec using deep-ultraviolet lithography. All of these are patterned via reactive ion etching, except for the microcomb layer of the bilayer microcomb, which is patterned using a damascene process. The integrated microcomb spectral filter is fabricated at NIST using electron-beam lithography and reactive ion etching.

## Supplementary information

Supplementary Information for Towards integrated photonic interposers for processing octave-spanning microresonator frequency combs
