# Supplementary material for: Towards integrated photonic interposers for processing octave-spanning microresonator frequency combs
Source: Light Sci Appl. 2021 May 26;10:109. doi: 10.1038/s41377-021-00549-y (PMC8155053; doi:10.1038/s41377-021-00549-y)
Supplement: Supplementary file 1 — Supplementary Information for Towards integrated photonic interposers for processing octave-spanning microresonator frequency combs [file 41377_2021_549_MOESM1_ESM.docx]

**Supplementary Information for**

**Towards integrated photonic interposers for processing octave-spanning microresonator frequency combs**

Ashutosh Rao,^1,2,∗^ Gregory Moille,^1,3^ Xiyuan Lu,^1,2^ Daron A. Westly,^1^ Davide Sacchetto,^4^ Michael Geiselmann,^4^ Michael Zervas,^4^ Scott B. Papp,^5,6^ John Bowers,^7^ and Kartik Srinivasan^1,3,†^

*^1^Microsystems and Nanotechnology Division, Physical Measurement Laboratory, National Institute of Standards and Technology, Gaithersburg, MD 20899, USA*

*^2^Maryland NanoCenter, University of Maryland, College Park, MD 20742, USA*

*^3^Joint Quantum Institute, NIST/University of Maryland, College Park, MD 20742, USA*

*^4^Ligentec, EPFL Innovation Park, Batiment C, Lausanne, Switzerland*

*^5^Time and Frequency Division, Physical Measurement Laboratory, National Institute of Standards and Technology, Boulder, CO 80305, USA*

*^6^Department of Physics, University of Colorado, Boulder, CO 80309, USA*

*^7^Department of Electrical and Computer Engineering, University of California, Santa Barbara, CA 93106, USA*

^∗^Email: [ashutosh.rao@nist.gov](mailto:ashutosh.rao@nist.gov)

^†^Email: [kartik.srinivasan@nist.gov](mailto:kartik.srinivasan@nist.gov)

**Note 1: Photonic platform**

Fig. S1: **Photonics Platform.** **a,b.** Optical confinement and number of modes for channel waveguides in a 400 nm thick silicon nitride film with silicon dioxide upper and lower cladding. The optical confinement here is defined as h = (∬_core_ |**E**(*x*,*y*)|^2^ d*x*d*y*)/(∬|**E**(*x*,*y*)|^2^ d*x*d*y*). A nominal waveguide width of 1 μm balances the confinement and number of modes across the octave, and is followed by additional tapering throughout the interposer to reach the target dimensions of specific elements (e.g., the dichroics). **c.** Waveguide transverse electric field modes simulated for wavelengths of 1 μm, 1.55 μm, and 2 μm, for a waveguide width of 1 μm. In keeping with **b**, |**E**(*x*,*y*)|^2^ is plotted.

Figures S1a and S1b show the variation of modal confinement of the fundamental transverse-electric (TE) mode and the number of TE modes with wavelength and waveguide width. Figure S1c shows simulated TE eigenmodes for a waveguide width of 1 μm that nominally balances these criteria. In selecting a photonic platform suitable for the proposed interposer, there are a few considerations that arise. The first is the ability to form the desired ring filters with free spectral ranges (FSRs) a little under 0.5 THz. This is related to the bending radius and optical confinement. The second is the efficiency of chip-to-chip coupling onto the interposer chip from lasers and microcomb chips. Finally, there is the footprint of the overall interposer, which is nominally limited by the heterogeneously integrated devices. In particular, for applications where 0.5 THz FSR ring filters are not essential, one can consider the use of low loss low-confinement nitride platforms where the core is approximately 100 nm thick and the optical mode significantly extends into the oxide cladding^1^. However, the microcomb pump filters here preclude our use of such a platform. Balancing the efficiency of chip-to-chip coupling with the other factors needs to be looked at on a case-by-case basis. Our use of a 400 nm thick silicon nitride film for the interposer devices and layer is suited to our proposed system. The use of other components and material systems may lead to a different optimal thickness. There are three instances of chip-to-chip coupling that occur into the proposed interposer (Fig. 2 of the main text and Fig. S8), with estimated coupling losses of < 1 dB across the octave from the THz microcomb chip, and <1 dB in the C-band from the GHz microcomb chip and the tunable laser chip, based on the mode overlaps. Here, the microcomb layer is 770 nm thick and can vary between 200 nm to 300 nm width at the facet using an oxide-clad inverse taper. The GHz microcomb consists of a silica microcomb coupled to a silicon nitride bus waveguide^2^ (250 nm by 900 nm at the facet, no top oxide cladding), and the tunable laser is based on the heterogeneous integration of III-Vs onto silicon-on-insulator^3^ (half-etched 500 nm by 5 μm oxide-clad ridge silicon cross section at the facet). Using a thicker device layer for the interposer will marginally increase coupling to the THz microcomb in practice but at the cost of decreasing the coupling to the GHz microcomb and tunable laser.

**Note 2: Multimode interferometers**

Figure S2 and Table S1 show the optimized design parameters of the multimode interferometers. Initial designs for a standard geometry^4^ were adapted and optimized for the butterfly geometry^5^ used here through 3D finite difference time domain (FDTD) simulations.


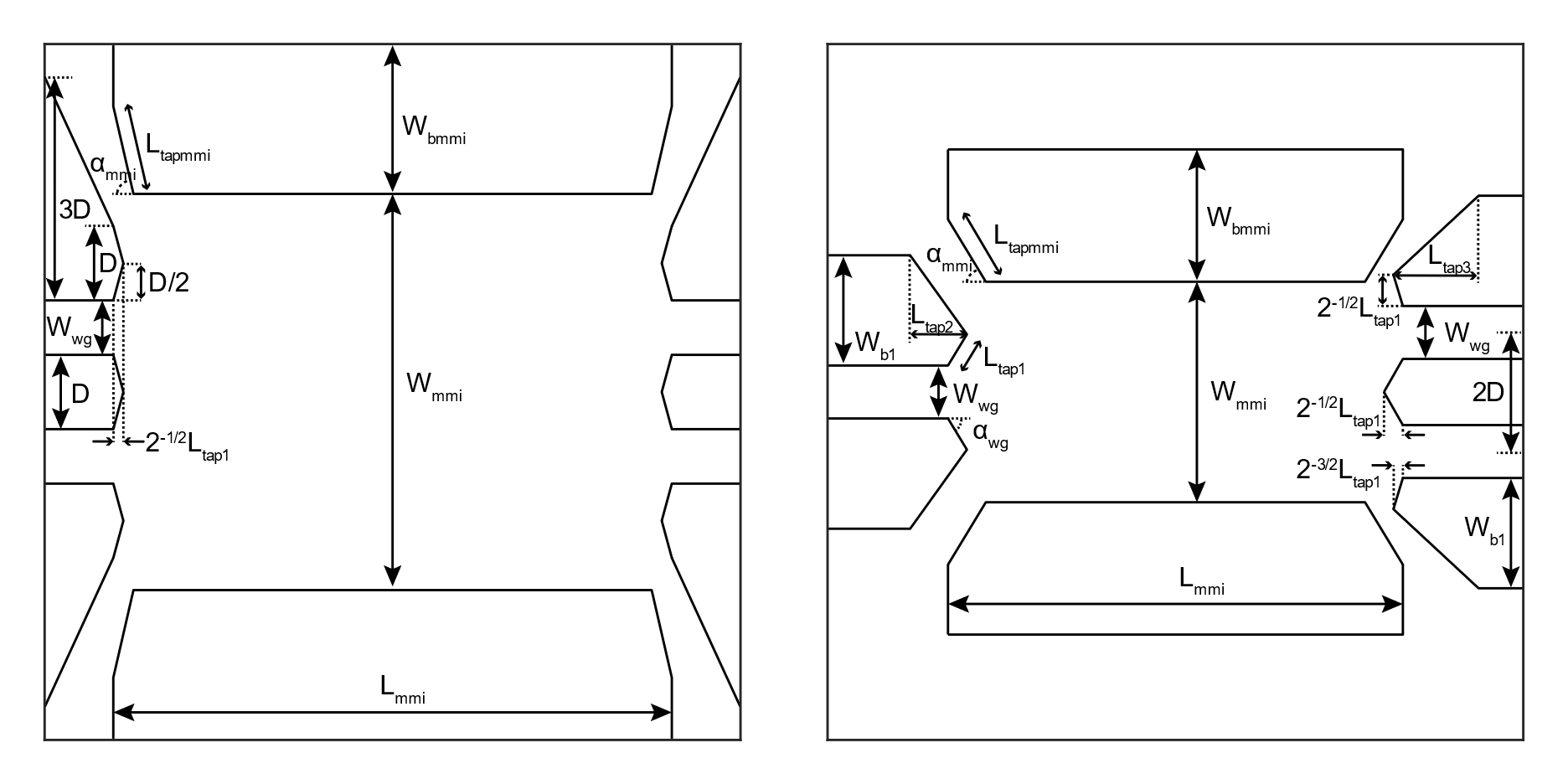


Fig. S2: **Multimode Interferometers.** Detailed design schematics for 2×2 and 1×2 multimode interferometers.

| **Parameter** | **2×2 1050 nm** | **2×2 1550 nm** | **1×2 1550 nm** |
| --- | --- | --- | --- |
| *L*_mmi_ (μm) | 23 | 49 | 15 |
| *W*_mmi_ (μm) | 7 | 8 | 5 |
| *W*_bmmi_ (μm) | 3 | 3 | 3 |
| *L*_tapmmi_ | 2*L*_tap1_ | 2*L*_tap1_ | 2 |
| *α*_mmi_ | 45° | 45° | 45° |
| *D* (μm) | 0.75 | 1.5 | 1.35 |
| *W*_wg_ (μm) | 1 | 1.1 | 1.2 |
| *α*_wg_ | - | - | 45° |
| *W*_b1_ (μm) | - | - | 2.5 |
| *L*_tap1_ (μm) | 1.25 | 1.25 | 1 |
| *L*_tap2_ | - | - | 3*L*_tap1_/$\sqrt{2}$ |
| *L*_tap3_ | - | - | 4.5*L*_tap1_/$\sqrt{2}$ |

Table S1: Geometrical parameters for multimode interferometers.

**Note 3: Microcomb pump ring filters**

The filter response depends on the intrinsic and coupling *Q*, as discussed in the main text. Figure S3 shows the variation of coupling *Q* with the coupling gap between the microring and bus waveguide, calculated using coupled mode theory^6^. The corresponding parameters used are ring radius = 50 μm, ring width = 1.5 μm, and bus waveguide width = 1 μm.


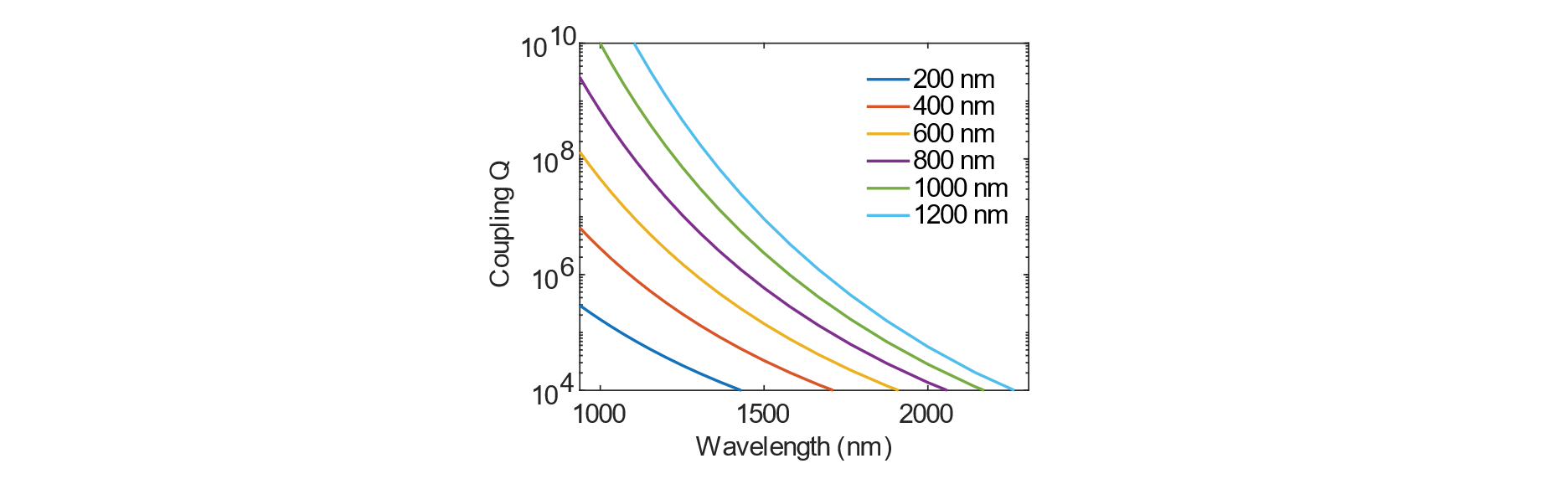


Fig. S3: **Ring filter coupling.** Simulated variation of ring filter coupling *Q* with coupling gap for a straight bus waveguide. The ring filter is meant to operate around 1550 nm wavelength. For a coupling *Q* ≈ 10^5^ at 1550 nm, the filter is severely undercoupled at 1000 nm wavelength, as desired, with coupling *Q* ≈ 5×10^7^.

**Note 4: Dichroic couplers**

The dichroic couplers (schematic shown in Fig. S4) used here are based on the strong dispersion, across the octave bandwidth, of the evanescent decay of the optical mode outside the waveguide core. Qualitative starting points for waveguide widths can be found in Fig. S1, which shows the optical confinement and is therefore indicative of the evanescent decay of the fundamental TE modes. Quantitatively, initial device parameters such as waveguide width and coupling gap are determined through finite element-method based eigenmode simulations of the supermodes of uniform couplers. The nominal coupling lengths extracted from these supermode simulations are used as starting points for 3D FDTD simulations that consider S-bends at the input and output of the dichroics. Table S2 shows the design parameters of the two optimized dichroics. Figure S5 shows the variation of dichroic coupler performance, extracted from continuous-wave measurements at wavelengths of 1.05 μm, 1.55 μm, and 2.05 μm. with coupling lengths, with optimal performance measured for the optimized designs.


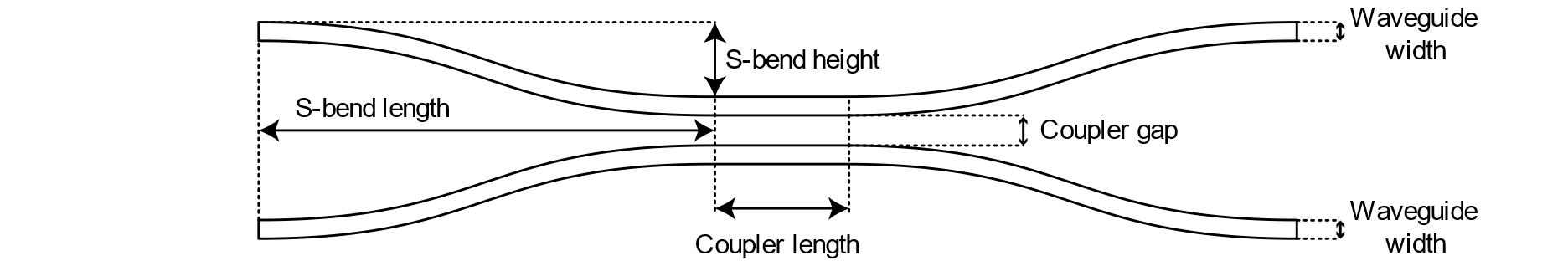


Fig. S4: **Dichroic couplers.** Schematic of dichroic couplers. Dichroic 1 filters out 2 μm light into its cross port, and dichroic 2 filters out 1.55 μm light into its cross port.

| **Parameter** | **Dichroic 1** | **Dichroic 2** |
| --- | --- | --- |
| Coupler length (μm) | 50 | 170 |
| Coupling gap (μm) | 1.25 | 2.5 |
| Waveguide width (μm) | 0.5 | 0.7 |
| S-bend length (μm) | 100 | 100 |
| S-bend height (μm) | 12.5 | 12.5 |

Table S2: Geometrical parameters (in μm) for dichroic couplers. Dichroic 1 filters out 2 μm light, and dichroic 2 filters out 1.55 μm light.


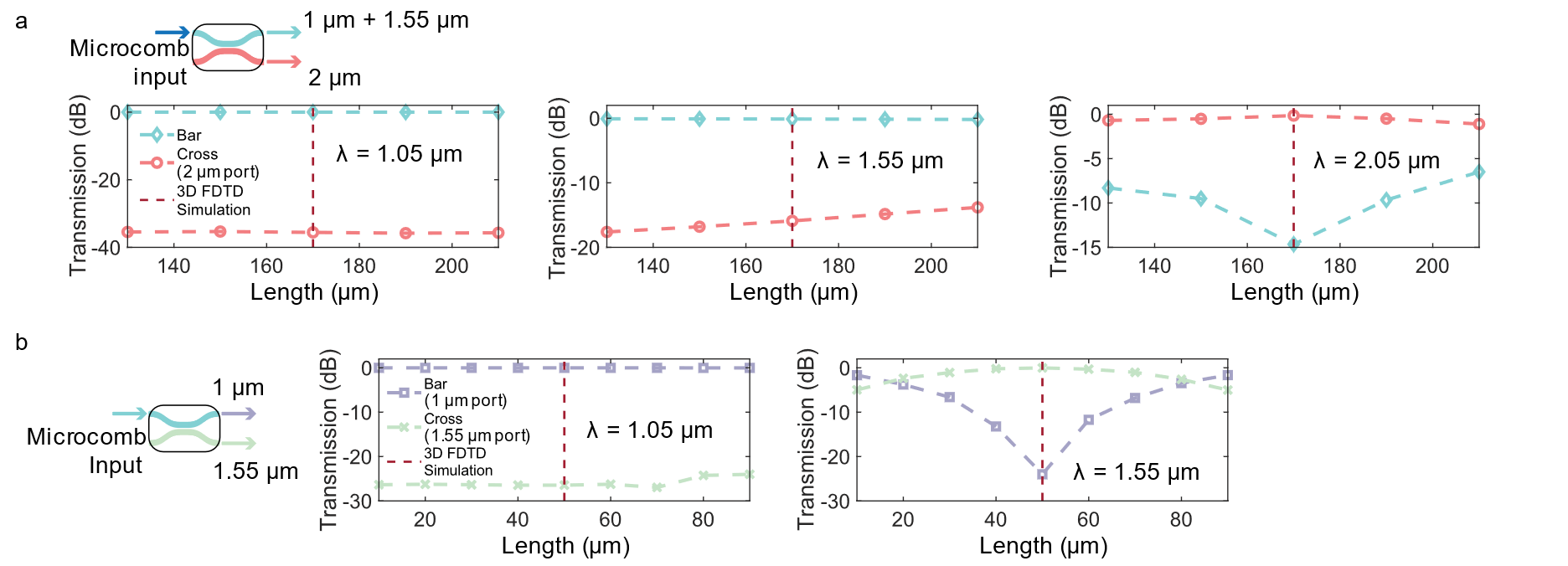


Fig. S5: **Continuous-wave measurements of dichroic couplers. a.** First dichroic, whose purpose is to separate 2 μm light from shorter wavelengths. **b.** Second dichroic, whose purpose is to separate 1.55 μm light from shorter wavelengths. Both dichroics offer optimal performance for coupling lengths that are in agreement with optimized FDTD simulations. The uncertainties in excess loss corresponding to one standard deviation in transmission are less than 0.25 dB at the three wavelengths.

**Note 5: Broadband bilayer taper**

Figure S6a shows a detailed schematic of the broadband bilayer taper. The transfer of light here requires a balance of the phasematching behind the bilayer coupling across the octave bandwidth. We limit the minimum widths of the tapers in accordance with the corresponding fabrication process (deep-UV lithography), and a broadband 3D FDTD sweep is used to determine the overall taper length. For a taper shorter than the optimal 100 μm, the bilayer coupling is reduced for shorter wavelengths close to 1 μm. Figure S6b shows the tolerance in taper transmission to interlayer thickness and taper misalignment.


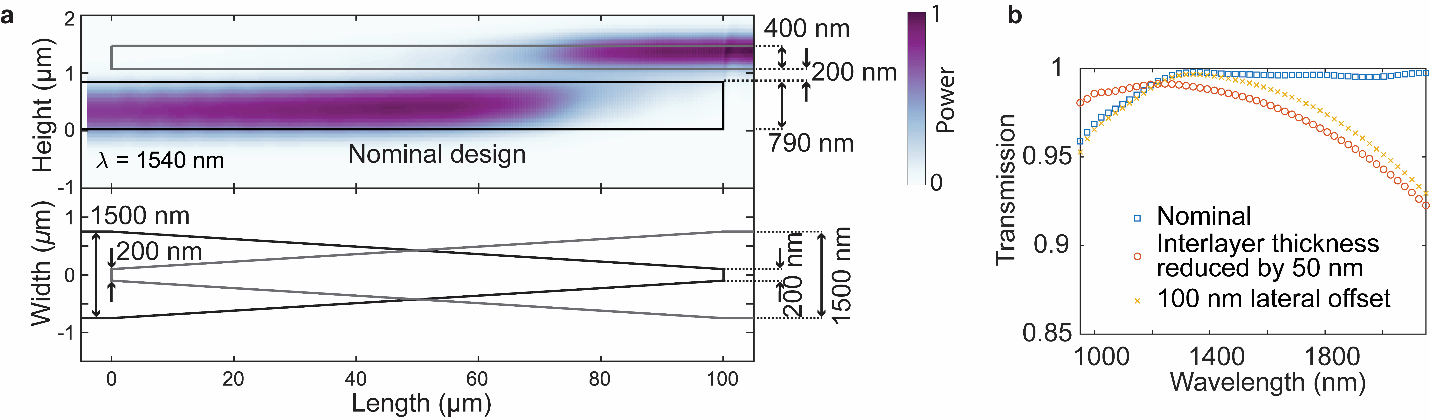


Fig. S6: **Bilayer coupler. a.** Cross section and top views of the bilayer coupler. Both layers are linearly tapered from 200 nm to 1500 nm. **b.** Transmission spectra for a reduced SiO_2_ interlayer thickness of 150 nm, and a 100 nm lateral misalignment between the tapers, compared to the nominal design.

A critical step to the successful realization of the bilayer platform in practice is planarization after fabrication of the thick Si_3_N_4_ layer, after which the interlayer silicon dioxide and interposer Si_3_N_4_ layers are deposited. Ellipsometry of process control wafers shows a mean interlayer SiO_2_ thickness of 204 nm (one standard deviation variation of 4 nm) and a mean top Si_3_N_4_ thickness of 401 nm (one standard deviation variation of 3 nm). While AFM measurements of the interlayer SiO_2_ surface roughness were not performed here, we note that a similar chemical-mechanical polishing process has been recently characterized^7^, and an SiO_2_ r.m.s. roughness <0.4 nm has been measured.

**Note 6: Experimental setups**

The experimental setups used are shown in Fig. S7, illustrating the different configurations used for measurements of the multimode interferometers, ring filters, dichroics, integrated spectral microcomb filter, and bilayer microcomb. Each continuous wave laser requires separate fiber components such as the 90:10 splitter and polarization controller, to satisfy the single mode criterion in the fiber. The detector following the 10 % port is used to assist in stabilizing the coupling to the device under test. TE polarization is used throughout all the measurements. Lensed optical fibers with focused spot sizes of ≈ 2.5 μm are used to couple light on and off the chips, aided by inverse tapers on the chips to match the mode profiles between the lensed fibers and waveguides.


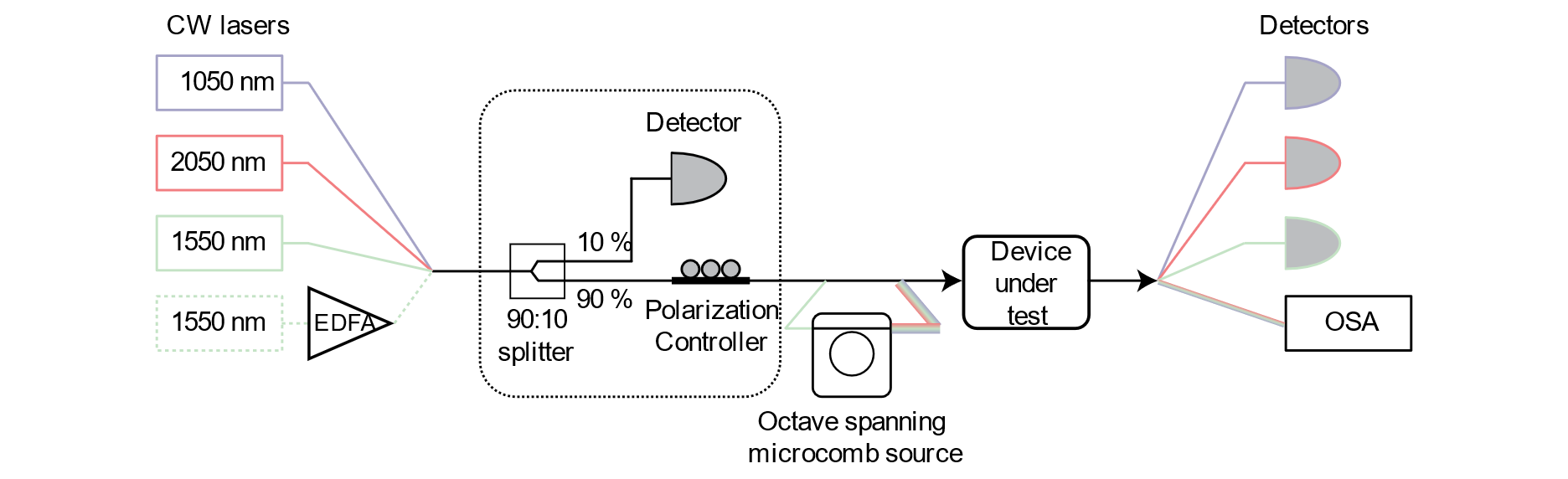


Fig. S7: **Experimental setups.** Multiple experimental setups are used throughout this work, depending on the combination of the device under test and the corresponding inputs. The devices tested are multimode interferometers, ring filters, dichroics, integrated spectral microcomb filter, and the bilayer microcomb. The inputs used are continuous wave lasers at 1050 nm, 1550 nm, and 2050 nm, and an octave spanning microcomb pumped around 1550 nm. Polarization maintaining fiber is used to couple the octave spanning microcomb to the corresponding devices under test. Two OSAs are used to cover the octave bandwidth of the microcomb. EDFA = Erbium-Doped Fiber Amplifier. OSA = Optical Spectrum Analyzer.

The measurement setup used to measure the dichroics with microcomb light consists of a polarization maintaining (PM) connection (2 lensed PM fibers connected by a-meter-long PM fiber using two fiber mating sleeves) between the source microcomb and the dichroics. The PM lensed fibers are rated for a polarization extinction ratio (PER) of 20 dB. The connecting PM fiber is rated for a PER of 25 dB. The two fiber mating connectors are not explicitly rated for PER. The PM fibers we use are 1550 nm XP fibers, rated for operation from 1440 nm to 1625 nm – much less than the octave of bandwidth we use here. We carefully minimize the bending of the fibers to minimize effects of polarization crosstalk and cut-off. Subsequently, our observations of deviations between the experiments and simulations below an extinction level around ≈ –20 dB (Figures 4 and 5 in the main text) are congruent with the PER of the setup. In comparison, the low power continuous wave measurements (Fig. S5) without the use of PM fiber show higher dichroic extinction compared to the microcomb measurements.

**Note 7: Summary of expected optical signal distribution in the proposed synthesizer**

Figure S8 and Table S3 together show how power would nominally be distributed in the proposed system in the three bands of interest (1 µm, 1.55 µm, and 2 µm). All chip-to-chip coupling losses are conservatively set to 2 dB. For completeness, we show all the elements required for a full system in Fig. S8 – continuous wave pump lasers and microcomb chips (silicon nitride for the THz comb and silica for the GHz comb), alongside the interposer proposed in Fig. 2 of the main text.


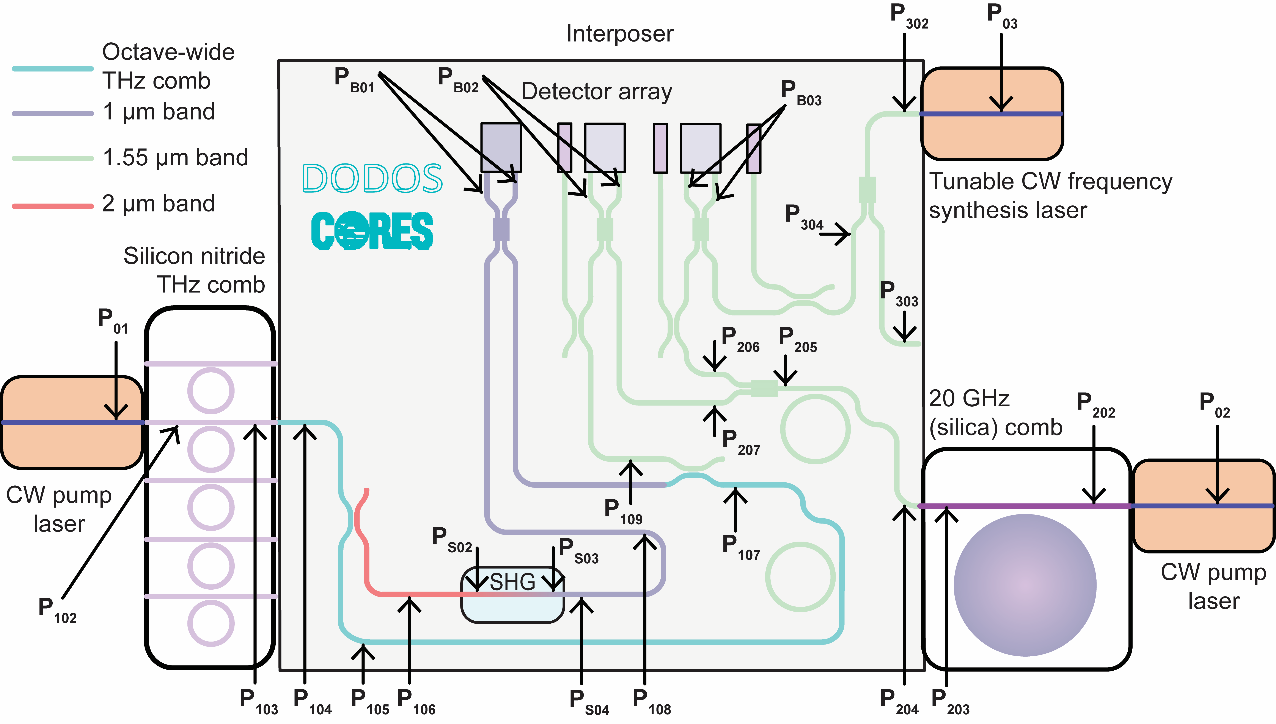


Fig. S8: **Power distribution schematic.** Schematic expanding on Fig. 2 of the main text. The labels showing power distribution throughout the proposed system correspond to Table S3. The schematic indicates how the passive components demonstrated here, i.e., octave-wide dichroics, tunable ring filters, and multimode interferometers, could fit into an interposer and system architecture for a dual-microcomb frequency synthesizer. Chip-to-chip coupled lasers and microcomb sources (silicon nitride and silica) form the dual-microcomb backbone. The THz repetition rate silicon nitride microcomb is used for *f*-2*f* self-referencing via a second harmonic generation-based frequency doubler. Dichroic directional couplers spectrally filter the 1 μm, 1.55 μm, and 2 μm bands of the silicon nitride microcomb. The 20 GHz repetition rate silica microcomb is used for repetition rate stabilization and as a reference for tuning the synthesized output laser. Throughout, multimode interferometers are used to mix signals to generated beat notes for frequency stabilization via balanced detection via fast photodetection. Additional photodetectors are used for power monitoring. In contrast to Fig. 2 of the main text, here the generic 20 GHz frequency comb is replaced by a silica microcomb to best correspond to our envisioned implementation. Alternatively, a 20 GHz silicon nitride microcomb could be considered as well.

| **Wavelength and power** | ***P*_01_  (dBm)** | ***P*_102_  (dBm)** | ***P*_103_ (dBm)** | ***P*_104_ (dBm)** | ***P*_105_ (dBm)** | ***P*_106_ (dBm)** | ***P*_107_ (dBm)** | ***P*_108_ (dBm)** | ***P*_109_ (dBm)** |  |
| --- | --- | --- | --- | --- | --- | --- | --- | --- | --- | --- |
| **1 µm** |  |  | -20/T | -22/T | -22/T |  | -22.3/T | -22.3/T |  |  |
| **1.55 µm** | 22 | 20 | -6/T | -8/T | -8/T |  | -8/T |  | -8/T |  |
| **2 µm** |  |  | -8/T | -10/T |  | -10/T |  |  |  |  |
| **Wavelength and power** | ***P*_S02_ (dBm)** | ***P*_S03_  (dBm)** | ***P*_S04_ (dBm)** | ***P*_02_  (dBm)** | ***P*_202_ (dBm)** | ***P*_203_ (dBm)** | ***P*_204_ (dBm)** | ***P*_205_ (dBm)** | ***P*_206_ (dBm)** | ***P*_207_  (dBm)** |
| **1 µm** |  | -35.3/T | -36/T |  |  |  |  |  |  |  |
| **1.55 µm** |  |  |  | 17 | 15 | -17/T | -19/T | -19/T | -22.2/T | -22.2/T |
| **2 µm** | -10.7/T |  |  |  |  |  |  |  |  |  |
| **Wavelength and power** | ***P*_03_  (dBm)** | ***P*_302_  (dBm)** | ***P*_303_ (dBm)** | ***P*_304_ (dBm)** | ***P*_B01_ (dBm)** | ***P*_B02_ (dBm)** | ***P*_B03_ (dBm)** |  |  |  |
| **1 µm** |  |  |  |  | -25.5/T &  -39.4/T |  |  |  |  |  |
| **1.55 µm** | 6.5 | 4.5 | 1.3 | 1.3 |  | -11.2&  -25.4/T | -1.9 &  -25.4/T |  |  |  |
| **2 µm** |  |  |  |  |  |  |  |  |  |  |

Table S3: Distribution of optical power throughout the proposed system. /T = per comb tooth

**Note 8: Discussion of proposed interposer and synthesizer architecture**

In the following note, we provide context for the values shown in Fig. S8 and Table S3. We address power requirements and transmission throughout the proposed system, starting with the lasers themselves, working our way through the microcomb chips, the passive interposer components, the second harmonic generation (SHG) section, the photodetectors, and ultimately end with power considerations for beat note signal-to-noise ratios (SNRs), with an aim to benchmark the performance required of the dichroics, ring filters, and MMIs. We also discuss additional system-level considerations when appropriate.

**Note 8.1: Microcomb power levels and compatibility with integrated lasers**

We first focus on the spectral power of the octave-spanning microcomb that underpins optical frequency synthesis applications. Figure S9 shows a THz microcomb generated in a Si_3_N_4_ microring at 100 mW of pump power in the waveguide. The microcomb spectrum is representative of what can be generated after careful optimization of microring dispersion^8,9^ and coupling^6^ for intrinsic quality factors around 2×10^6^. For 2 dB of coupling loss between the microcomb chip and a chip-scale laser, the laser power requirement is around 160 mW, which is achievable from integrated lasers^10^. Dispersive waves at *f* and 2*f* frequencies help to boost the carrier envelope offset frequency (*f*_CEO_) signal.


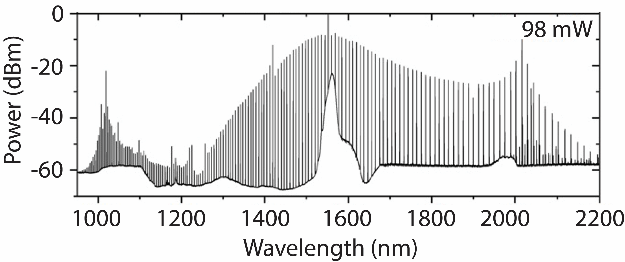


Fig. S9: **Representative THz comb.** This comb is generated using 98 mW of pump power in the Si_3_N_4_ waveguide.

It has been shown that C-band-spanning comb generation at a 10 GHz to 20 GHz rep rate can be realized with ~30 mW of on-chip pump power, either from silica-based combs^11^ or silicon nitride combs^12^. Using a conservative estimate of 2 dB of coupling loss amounts to a power requirement of ~ 50 mW from a chip laser, which is within the performance of chip lasers.

One key consideration common to self-referenced microcomb-based systems is the pinning down of the carrier envelope offset frequency (*f*_CEO_) of the microcomb into the bandwidth of the photodetector using for *f*-2*f* self-referencing. In the context of the system proposed in Fig. 2 of the main text and Fig. S8 above, this translates to pinning down a THz bandwidth of potential *f*_CEO_ variation into the nominal 10 GHz bandwidth of the balanced photodetectors. Keeping in mind the ability to prequalify the octave-spanning THz microcomb in schemes like Fig. 2 (where the THz comb chip is separate from the interposer chip), there are a few approaches to managing f_CEO_ appropriately, where *f*_CEO_ can be measured before system assembly. The first method uses a parametric sweep of the microring radius and width to realize a sweep of *f*_CEO­_, with approximate tuning rates of 1 GHz/nm and -8 GHz/nm, respectively, for the THz Si_3_­N_4_ microcombs considered here. Sweeping the microring radius is preferred due to its minimal impact on the microring dispersion (and hence the generated comb spectrum). The second relies on thermal tuning, where previously 25 GHz tuning of *f*_CEO_ has been shown in a 231 GHz repetition rate Si_3_­N_4_ microcomb for a 70°C rise in temperature^13^. Finally, the third is based on post-fabrication trimming of air-clad resonators, which has been previously employed to adjust resonance frequency mismatch and dispersion for four-wave mixing and microcombs^14,15^. From a practical standpoint, it is possible to pattern over 300 microcomb resonators within a 3×5 mm^2^ chip, which is sufficient to constrain the 1 THz *f*_CEO_ variation to the 10 GHz photodetector bandwidth. With sufficient fabrication process control, it should be feasible to eventually monolithically integrate an octave-spanning THz microcomb with appropriate *f*_CEO_ using a bilayer scheme as shown in Fig. 6 of the main text, particularly if some *in-situ* control (e.g., the thermal tuning) is available.

**Note 8.2: First dichroic (2 µm band separation) inhibits two-photon absorption in the gallium arsenide based second harmonic generation waveguide**

An integrated synthesizer would require a SHG section that interfaces with a passive interposer. Here, we consider a gallium arsenide (GaAs) based waveguide that can be heterogeneously integrated^16,17^ onto the interposer. In this context, one consideration for the first dichroic element that operates on the THz comb is to avoid the potential of strong pump light at 1550 nm causing damage in the expected GaAs SHG element mediated by two-photon absorption (2PA). We use *P*_max_ to denote the ~ 100 mW pump power. For degenerate 2PA, the 2PA coefficient (*β*) around 1.55 μm in GaAs is approximately 5 cm/GW^18^. Approximating the modal area by the area of the GaAs waveguide^17^ (*A*_wg_), 150 nm × 1900 nm, the maximum possible 2PA in the SHG section (ignoring all coupling losses) scales as (*βP*_max_/*A*_wg_)×(*ER*_D1,1.55µm_) which is approximately 0.76×(*ER*_D1,1.55µm_) dB/cm. Here, *ER*_D1,1.55µm_ is the extinction ratio of the 1^st^ dichroic at 1.55 μm. Given that our 1st dichroic has approximately 20 dB of extinction (i.e., *ER*_D1,1.55µm_ = 0.01) in the 1.55 μm band, we can rule out any damage and refraction induced by degenerate 2PA. In addition, although non-degenerate 2PA is more complicated to analyze and wavelength-dependent absorption coefficients are not readily available in the literature, the fact that the next-strongest microcomb teeth are weaker in power by 15 dB to 20 dB or more compared to the pump tone implies that non-degenerate 2PA is unlikely to play any significant role.

**Note 8.3: Transmission of 2 µm band and SHG power generated in the 1 µm band**

Next, we consider the SHG section, comprised of a taper from Si_3_N_4_ to GaAs, followed by type I SHG in the GaAs waveguide (2 µm TE input and doubled 1 µm TM output), and an asymmetric taper/rotator to transfer light back to the Si_3_N_4_ and to rotate the doubled 1 µm TM light to TE polarization. The heterogeneous integration of GaAs and Si_3_N_4_ for our proposed system has been previously reported^16,17^. Within fabrication tolerances, both the input taper and output taper/rotator transmission and rotation efficiency are expected to be >80%, with nominal values of >95% and 90%, respectively^16^. Efficient SHG in a GaAs-on-insulator waveguide (without Si_3_N_4_ integration) for our proposed system has been reported^17^ with a SHG efficiency of 40 W^-1^ (i.e., 4000%/W). Using this nonlinear efficiency, and a conservative estimate of 85% for both Si_3_N_4_/GaAs transitions, we expect a frequency doubled power of -36 dBm in the 1 µm band.

**Note 8.4: Transmission of 1 µm band**

The 1 µm band of the THz microcomb traverses the first dichroic, the resonant pump filter, and the second dichroic. The extinction ratio through the first dichroic is approximately > 20 dB, i.e., <1% of the light is rejected. The spectral alignment of the resonant pump filter with the pertinent 1 µm microcomb tooth to be used to measure *f*_CEO_ is difficult to precisely predict a priori, however, we can calculate the maximum loss possible when the pump filter and comb tooth are perfectly aligned. For a coupling *Q* of 5x10^7^ (see Fig S3), the maximum possible transmission loss is 0.3 dB at 1 µm, calculated using analytic coupled mode theory for an add-drop microring^19^ using an intrinsic *Q* of 1 million. Finally, the second dichroic that extracts 1.55 µm light has an extinction ratio of > 20 dB, i.e., < 1% loss. Cumulatively, we expect > 90% transmission in the worst case of the unwanted alignment between the ring filter and the 1 µm microcomb tooth used for f_CEO_.

**Note 8.5: Transmission of 1.55 µm band**

Next, we consider the 1.55 µm band of the THz comb. The first dichroic shows 20 dB of extinction. The extinction offered by the pump filter is tunable and its spurious spectral overlap with the remainder of the THz comb in the *C*-band is alleviated by an intrinsic vernier effect between the THz repetition rate and 478.4 GHz filter FSR. The second dichroic shows 18.5 dB of extinction, implying approximately 97% overall transmission (0.1 dB loss).

**Note 8.6: Photodetectors and transimpedance amplifiers:**

Finally, we need to consider the performance of the photodetectors and transimpedance amplifiers (TIAs) to quantify the role of the performance of the passive interposer components at a system level. Photodetectors heterogeneously integrated on Si_3_N_4_ (without TIAs) suited for our system have been reported^20^ with responsivities of 0.83 A/W and 0.94 A/W in the 1 µm and 1.55 µm bands, respectively. Balanced photodetectors show common mode rejection ratios > 40 dB and bandwidths of 10 GHz. Single photodetectors show bandwidths of 20 GHz and dark currents of 20 nA, sufficient to directly detect the repetition rate of the 20 GHz silica microcomb with SNR well in excess of 30 dB. In addition, photodetectors integrated on a printed circuit board with TIAs (no heterogeneous integration with Si_3_N_4_) have been reported^21^ where two designs show bandwidths around 10 GHz, maximum conversion gains between 1289 to 2083 V/W, and minimum noise equivalent powers (NEPs) of 13 pW/$\sqrt{\mathrm{Hz}}$. Details regarding recent progress in the TIAs can be found in Ref. 22. Furthermore, a heterodyne receiver-based approach to tackle potential SNR limitations in optical comb power was previously reported in Ref. 23.

**Note 8.7: Pump laser extinction ratios**

The main consideration in determining the extinction applied to the pump lasers by the tunable ring filters is the need to not saturate, or worse, damage, the photodetectors. At the same time, there is a question of how much extinction is sufficient and can let the rest of the system operate unimpeded. Our approach has been to filter the pump to match the adjacent comb teeth – for the THz comb, this power level is intrinsically compatible with the subsequent dichroic filtering (at the 2^nd^ dichroic) prior to beat note detection. For the 20 GHz comb, this level of pump filtering also avoids an excessive dynamic range requirement when measuring the beat note between the 20 GHz comb and the tunable synthesis laser. On the other hand, if, for example, the 20 GHz comb pump was entirely filtered out (with say 100 dB of extinction), there would be a discontinuity between comb teeth around the pump, and consequently also in the tuning range of the synthesis laser that is referenced to the 20 GHz comb.

**Note 8.8: Beat note SNRs**

There are three beat notes to be detected for stabilization and synthesis – the carrier envelope offset frequency *f*_CEO_, the inter-comb beat note for locking the two combs (hereafter referred to as dual comb lock (DCL) for brevity), and the offset between the tunable laser and the silica comb (hereafter referred to as tunable laser lock (TLL) for brevity) for synthesis. The 20 GHz repetition rate of the silica microcomb is directly detected, as discussed in Note 8.6. In the following estimates of the SNRs of these three beat notes, we use a conservative NEP of 20 pW/$\sqrt{\mathrm{Hz}}$ (see Refs. 20 and 21) and optical powers and system parameters as shown in Fig S8 and Table S3. First, we consider the variation of the CEO SNR with the detection bandwidth, shown in Fig. S10, which illustrates a tradeoff – between the high SNR offered by low detection bandwidths and the low SNR caused by the need for higher bandwidths required to operate a standalone system. Using a broadband radiofrequency (RF) mixer and a swept intermediate frequency (IF) for RF down-conversion followed by a narrow low pass filter (LPF) can offer an intermediate resolution to this tradeoff, with a nominal 5 MHz of bandwidth (from the low pass filter) and corresponding 16.9 dB of CEO SNR. If we relax the conservative coupling loss estimates to 1 dB from 2 dB, we expect the CEO SNR to increase to 25.5 dB, based on increases in microcomb power and increased SHG. The DCL SNR and TLL SNR are estimated to be 25 dB and 31.1 dB, respectively, when both detected using 50 MHz bandwidth using a similar swept IF RF downconversion with a LPF^23^.


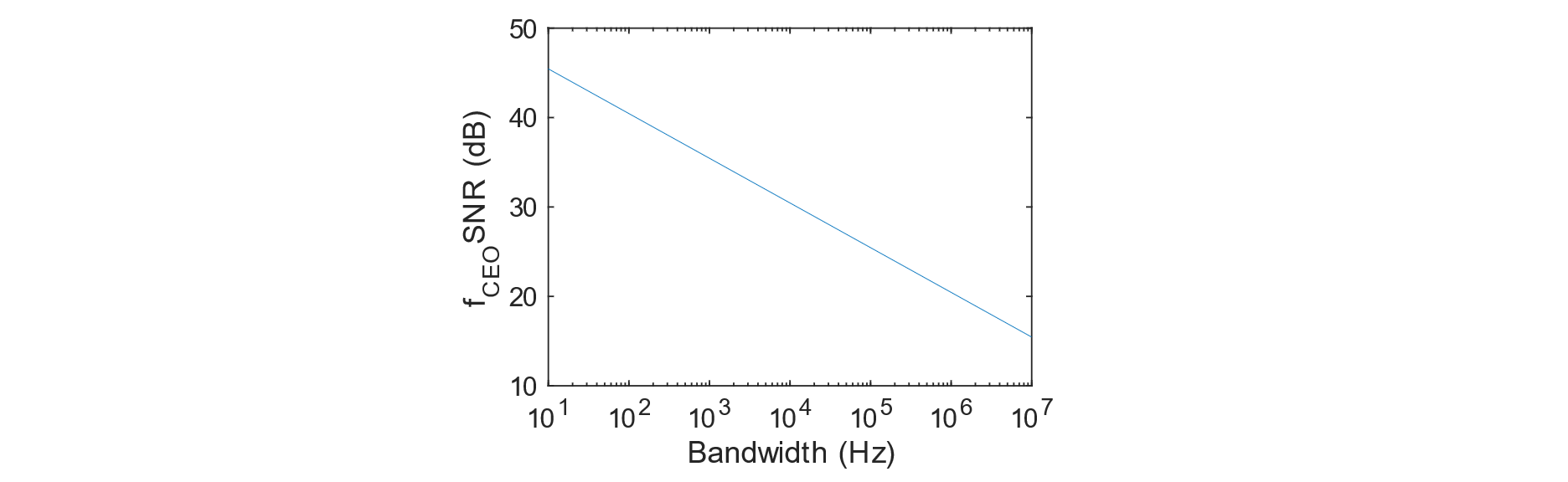


Fig. S10: **Calculated signal-to-noise ratio of carrier envelope offset frequency in proposed synthesizer**. Variation with detection bandwidth.

**Note 8.9: Impact of dichroic and MMI performance on beat note SNRs:**

Figure S11 shows the impact of the dichroics’ performance on the CEO and DCL SNR. The TLL SNR is unaffected by the dichroics. For the CEO SNR calculation, we assume that the extinction ratios for the 1 µm band are the same at the 1st and 2nd dichroic, for ease of representation. 3 dB of extinction would imply 50:50 splitting.


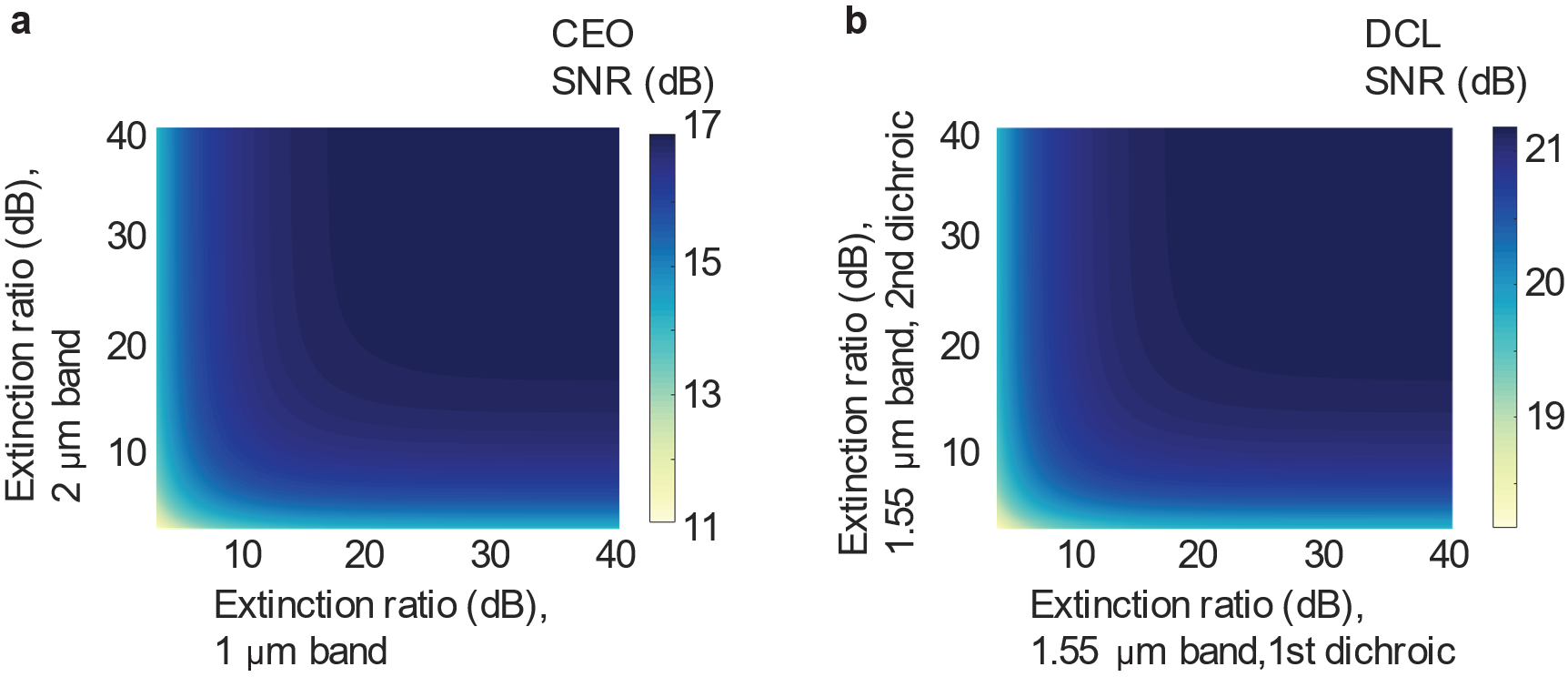


Fig. S11: **Calculated signal-to-noise ratio of carrier envelope offset frequency and dual comb locking in proposed synthesizer**. Variation with dichroic performance.

While significantly worse dichroic extinction would decrease the CEO and DCL SNRs to different extents, an increase in the dichroic extinction ratios even up to 40 dB from the current ~ 20 dB will have comparatively negligible improvement in the SNRs. Thus, the current performance of the passive interposer components would not be the limiting factor in increasing the CEO and DCL SNRs. Other factors, such as increasing the THz pump power to its maximum of 250 mW^10^, increasing the SHG efficiency, and decreasing the NEP of the photodetectors, would have to drive increases in SNR. Overall, this is reasonable when considering the excellent progress in integrated photonics, for example, SHG efficiencies have steadily increased across different material systems. Outside of the photonic devices, reducing the electronic bandwidth after detection will improve SNR, as discussed in Note 8.8.

The MMIs have two functions in the proposed interposer system - the first is to split power (1x2 MMIs), and the second is coherent mixing (2x2 MMIs). While only the 1 µm band 2x2 MMI is used for the CEO beat note, the beat notes for dual comb locking and tunable laser locking involve 2 and 3 MMIs in the 1.55 µm band. The impact of MMI excess loss (Fig. 3b, main text) on the beat note SNRs is shown in Fig. S12.


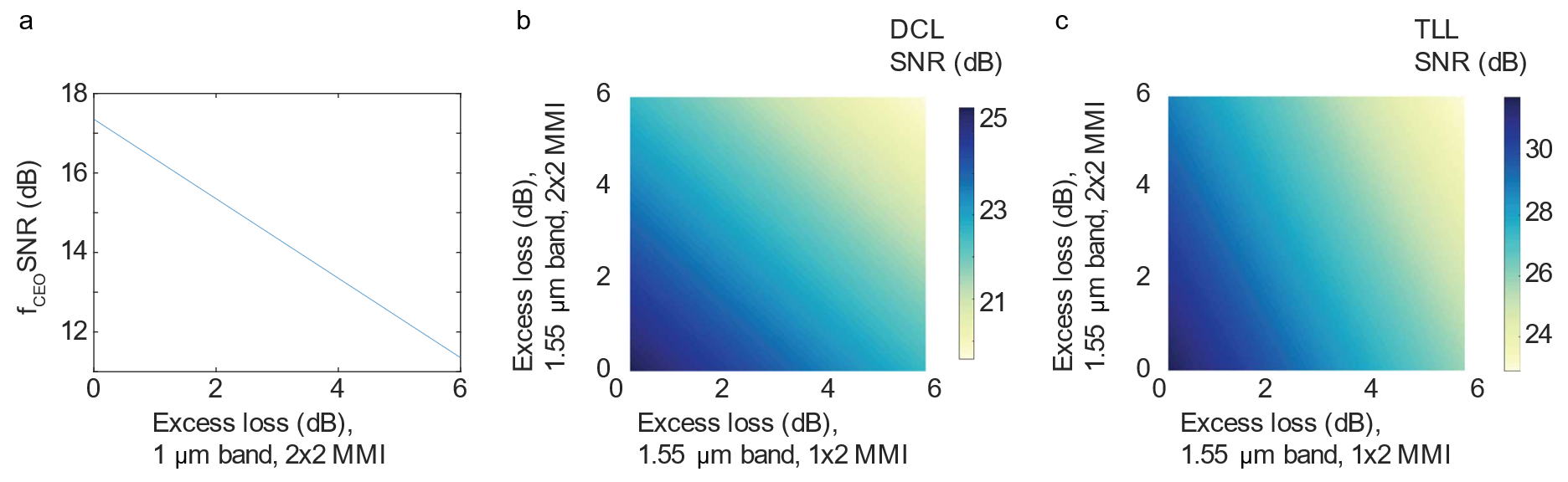


Fig. S12: **Calculated signal-to-noise ratio of carrier envelope offset frequency, dual comb locking, and tunable laser locking for the proposed synthesizer**. Variation with MMI performance.

Similar to the dichroics, any improvement in the demonstrated excess loss of the MMIs would lead to only a small increase in beat note SNRs, while an increase in overall system optical power and transmission, and a decrease in photodetector noise would improve the beat note SNRs significantly.

**References**

1. M.-C. Tien et al, “Ultra-low loss Si_3_N_4_ waveguides with low nonlinearity and high power handling capability,” Opt. Express **18**, 23562-23568 (2010).
2. K. Y. Yang et al, “Bridging ultrahigh-Q devices and photonic circuits,” Nat. Photon. **12**, 297–302 (2018).
3. M. A. Tran, D. Huang, and J. E. Bowers, “Tutorial on narrow linewidth tunable semiconductor lasers using Si/III-V heterogeneous integration,” APL Photonics 4, 111101 (2019).
4. L. B. Soldano and E.C. Pennings, “Optical Multi-Mode Interference Devices Based on Self-Imaging: Principles and Applications,” Journal of Lightwave Technology **13**, 615–627, (1995).
5. E. Kleijn et al, “Multimode interference couplers with reduced parasitic reflections,” IEEE Photonics Technology Letters **26**, 408–410 (2014).
6. G. Moille et al, “Broadband resonator-waveguide coupling for efficient extraction of octave-spanning microcombs,” Optics Letters **44**, 4737–4740 (2019).
7. L. Chang et al, “Heterogeneous integration of lithium niobate and silicon nitride waveguides for wafer-scale photonic integrated circuits on silicon,” Opt. Lett. **42**, 803–806 (2017).
8. Q. Li et al, “Stably accessing octave-spanning microresonator frequency combs in the soliton regime,” Optica **4**, 193-203 (2017).
9. T. C. Briles et al, “Interlocking Kerr-microresonator frequency combs for microwave to optical synthesis,” Opt. Lett. **43**, 2933–2936 (2018).
10. <https://freedomphotonics.com/freedom-photonics-products/lasers-and-laser-sources/1550-nm-high-power-dfb-laser-fp3815/>
11. X. Yi, Q.-F. Yang, K. Y. Yang, M.-G. Suh, and K. Vahala, "Soliton frequency comb at microwave rates in a high-Q silica microresonator," Optica **2**, 1078-1085 (2015).
12. J. Liu et al, “Photonic microwave generation in the x-and k-band using integrated soliton microcombs,” Nat. Photonics **4428**, 1–6 (2020).
13. X. Xue et al, “Thermal tuning of Kerr frequency combs in silicon nitride microring resonators,” Opt. Express **24**, 687-698 (2016).
14. X. Lu et al, “Efficient telecom-to-visible spectral translation through ultralow power nonlinear nanophotonics,” Nat. Photonics **13**, 593–601 (2019).
15. G. Moille et al, “Post-Processing Dispersion Engineering of Frequency Combs in Microresonator Addressing Atomic Clock,” in *Conference on Lasers and Electro-Optics*, OSA Technical Digest (Optical Society of America, 2020), paper SW3J.5.
16. E. J. Stanton et al, “On-chip polarization rotator for type I second harmonic generation,” APL Photonics **4**, 126105 (2019).
17. E. J. Stanton et al, “Efficient second harmonic generation in nanophotonic GaAs-on-insulator waveguides,” Optics Express **28**, 9521-9532 (2020).
18. W. C. Hurlbut et al, “Multiphoton absorption and nonlinear refraction of GaAs in the mid-infrared,” Optics Letters **32**, 668-670 (2007).
19. C. Manolatou et al, “Coupling of modes analysis of resonant channel add-drop filters,” IEEE Journal of Quantum Electronics **35**, 1322–1331 (1999)
20. Q. Yu et al, “Heterogeneous photodiodes on silicon nitride waveguides,” Opt. Express **28**, 14824-14830 (2020).
21. R. Costanzo, Z. Yang, A. Beling, and S. M. Bowers, “Wideband Balanced Photoreceivers With InP-Based Photodiodes and 65 nm CMOS TIAs for Use in Optical Frequency Synthesis Systems,” Journal of Lightwave Technology **37**, 5833 - 5839 (2019).
22. R. Costanzo and S. M. Bowers, “A 10-GHz Bandwidth Transimpedance Amplifier With Input DC Photocurrent Compensation Loop,” IEEE Microwave and Wireless Components Letters **30**, 673-676 (2020).
23. A. Bluestone et al, “Heterodyne-based hybrid controller for wide dynamic range optoelectronic frequency synthesis,” Opt. Express **25**, 29086-29097 (2017).
